# AI‐Physics‐Experiment Trinity for Integrated Protein Dynamics Modeling

**DOI:** 10.1002/advs.76023

**Published:** 2026-06-16

**Authors:** Chen Shi, Minying Low, Peng Xiu, Kresten Lindorff‐Larsen, Yong Wang

**Affiliations:** ^1^ College of Life Sciences & Department of Engineering Mechanics Zhejiang University Hangzhou China; ^2^ Linderstrøm‐Lang Centre for Protein Science Department of Biology University of Copenhagen Copenhagen Denmark; ^3^ The Provincial International Science and Technology Cooperation Base on Engineering Biology International Campus of Zhejiang University Haining Zhejiang China

**Keywords:** generative AI, integrative structural biology, MD simulations, protein dynamics, protein ensemble modeling

## Abstract

Proteins exist as conformational ensembles, with dynamic transitions governing biological processes. Deciphering these dynamics demands integrating experimental data, physics‐based simulations, and artificial intelligence (AI)—each with distinct strengths and limitations. Experiments deliver direct structural and dynamic benchmarks but are often constrained by insufficient spatiotemporal resolution or difficulties with providing information on transiently and weakly populated states. Physics‐based methods may generate atomic‐scale trajectories via force fields yet face sampling bottlenecks, force field sensitivity, and the curse of dimensionality. AI, particularly deep learning and generative modeling approaches, facilitates the efficient prediction of protein structures and conformational ensembles, as well as dimensionality reduction, yet is hindered by limited interpretability and transferability, and a scarcity of high‐quality ground‐truth data for training and benchmarking models of dynamics. This review outlines core principles of standalone approaches and highlights integrative strategies: experimental constraints guide physics‐driven refinement; AI enhances experimental processing and ensemble generation; physics imparts plausibility to AI, while AI accelerates simulation sampling and force field optimization. We elaborate on this synergy, emphasizing physics‐based modeling's glue‐like role in reconciling heterogeneous datasets. Finally, we summarize persistent challenges and discuss future directions for integrated modeling of protein dynamics.

## Graphical abstract

This review unites experiments, physics‐based simulations, and AI as a synergistic triad for protein dynamics modeling. It highlights integrative strategies, resolves sampling and forcefield bottlenecks, and outlines challenges and future directions for accurate, interpretable conformational ensemble prediction.

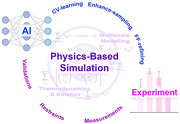

## 1 Introduction

Proteins are the central executors of biological processes. A fundamental paradigm in modern protein biology is the recognition that proteins exist as conformational ensembles rather than a single “static” structure [1, 2, 3]. Nearly all protein‐mediated processes, spanning enzyme catalysis, ligand binding, signal transduction to molecular transport, rely on transitions between conformational states, each displaying intra‐state motions [4]. Thus, unraveling the principles governing protein structural dynamics has become a central objective, with computational approaches playing a pivotal role in bridging theoretical models and experimental observations [5].

**FIGURE 1:**
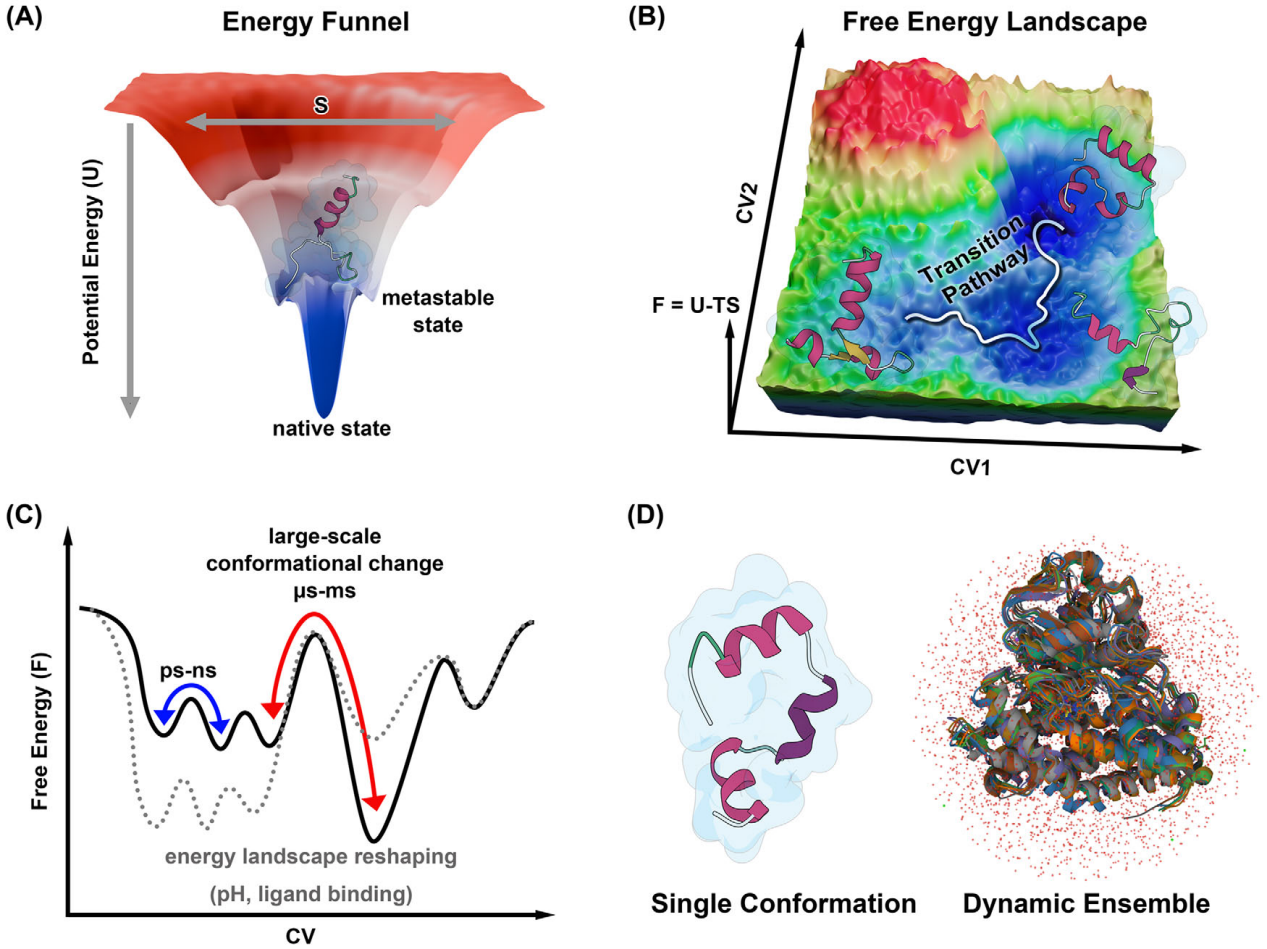
The protein energy landscape paradigm for integrated modeling. (A) The energy funnel, where the vertical axis represents the potential energy (U) of the protein. As a protein folds, it navigates a rugged landscape toward the global minimum corresponding to the native state. The width of the funnel at any given energy level, denoted as S, represents the configurational entropy. Local minima along the funnel correspond to metastable states or kinetic traps that the protein may transiently populate during the folding process. Note that while potential energy drives the structural collapse, the actual thermodynamic stability is determined by the free energy, as shown in panel (B). (B) A free energy landscape (F = U – TS) projection, colored by stability (red = high energy, blue = low energy), shows a transition pathway between states, defined by collective variables (CVs). (C) A free energy landscape slice illustrates rugged topography, including fast (ps–ns, blue) and slow (µs–ms, red) conformational transitions, and landscape reshaping from cellular perturbations (e.g., temperature, pH, ligand binding, etc.). (D) Contrast of the historical static “single conformation” view (left) and modern dynamic “ensemble” paradigm (right), which motivates integrating experiments, physics, and AI to capture functional protein dynamics.

At the molecular level, protein conformations (spatial 3D structural shapes) are governed by the potential energy surface (PES), where each point maps to a specific conformation and its associated potential energy. This potential energy is determined by intramolecular interactions (e.g., hydrogen bonds, hydrophobic interactions, electrostatic forces) and intermolecular interactions with solvent (e.g., water molecules) or other biomolecules [6, 7]. While the potential energy describes the energetic state of a single conformation, biological processes are governed by the free energy, integrating potential energy and entropy. The Boltzmann distribution quantifies the population of each conformational state at a given temperature: the probability of a protein occupying a specific conformation is inversely proportional to the exponential of its potential energy relative to the thermal energy (k_B_T) [8]. Conformations with lower free energies are thus more populated (and thus easier to capture in experiments and simulations), while higher‐energy transient conformations, though less abundant, can be biologically critical and pose significant challenges for experimental measurements and computational sampling [9]. The free energy landscape (FEL) emerges from the statistical projection of the high‐dimensional PES onto a small number of collective variables, often chosen to represent slow motions and after thermodynamic averaging over fast atomic and solvent fluctuations, providing a thermodynamical representation of protein dynamics. It highlights low‐free‐energy basins and, potentially, the energy barriers between them, which govern the rate of conformational transitions and are the focus of kinetic modeling in computational studies (Figure 1) [8, 10].

Rooted in statistical mechanics, the ensemble concept provides the theoretical foundation for computational simulations: a protein sample (or a single protein over time) populates a set of conformational states, each with a characteristic probability defined by the Boltzmann distribution. This stands in contrast to the historical rigid “lock‐and‐key” model of enzyme‐substrate binding [11]. Protein dynamics encompasses temporal fluctuations within this ensemble across multiple timescales: small‐scale motions (e.g., side‐chain rotations, backbone fluctuations, loop motions) on picosecond to nanosecond timescales and large‐scale conformational transitions (e.g., domain motions, folding/unfolding) on microsecond to millisecond or longer timescales [4, 12].

The integration of statistical mechanics and MD simulations has facilitated the modeling of protein dynamics, yet significant computational challenges remain. Foremost among these is the high dimensionality of protein conformational space (the set of all 3D structural states) —characterized by 3N degrees of freedom (where N is the number of atoms in the system)—which renders explicit enumeration of all microscopic states computationally intractable [13]. This limitation restricts direct calculation of the partition function(a statistical mechanical sum over all accessible microscopic states of a system that directly encodes thermal fluctuations) and free energy for large proteins [14]. Furthermore, while experimental techniques, such as nuclear magnetic resonance (NMR) spectroscopy for ensemble‐averaged and site‐specific dynamic information, and X‐ray crystallography and single‐molecule cryogenic electron microscopy (cryo‐EM) for individual conformational states, provide critical data, each has inherent limitations in capturing the full scope of conformational ensembles (collections of interconverting structural states). No single experimental or computational approach can independently furnish a comprehensive conformational, thermodynamic, and kinetic description. Thus, integrating heterogeneous data sources into computational frameworks represents a practical strategy, as experimental data alone cannot fully constrain the conformational landscape (the full energetic and structural space of protein conformational transitions). This underscores the necessity of integrated strategies that combine experimental constraints with computational modeling to address these gaps.

**FIGURE 2:**
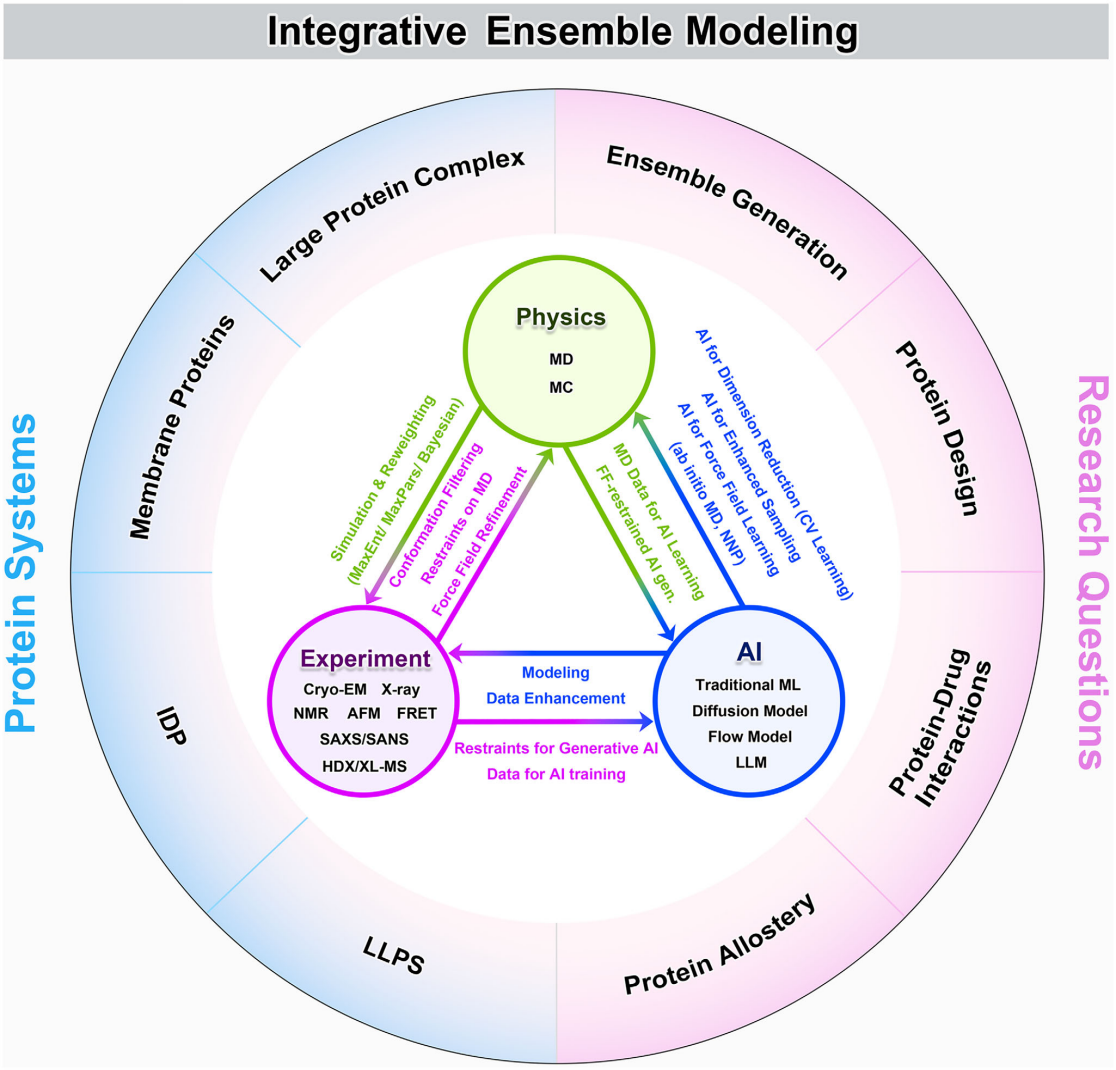
Integrative Ensemble Modeling framework, which unites experiments, physics‐based simulations, and AI in an integrated framework. Experiments provide structural data (e.g., cryo‐EM, NMR) and validation for model outputs, physics uses force field based molecular dynamics (MD) and Monte Carlo (MC) simulations to sample conformational ensembles and refine AI models, and AI methods employ machine learning (ML), diffusion models, and large language models (LLMs) etc. to generate and analyze ensembles, enhance sampling, and predict structures, all to address diverse research questions (e.g., protein conformational changes, drug interactions) across a range of protein systems (e.g., large protein complex, membrane proteins, intrinsically disordered proteins (IDPs) and biomolecular condensates).

Notably, force field‐ or other physics‐based simulation methods may serve as an important bridge in both processes [15]. They enable the translation of experimental constraints into quantitative models of protein energetics and dynamics, forming the core of integrative structural biology from a computational perspective. This is where the convergence of experiments, physics (statistical mechanics), and AI has emerged as an increasingly important framework. Experimental data serve as critical constraints to focus simulations to sample the most relevant regions of the conformational space; statistical mechanics provides the theoretical framework to model the energetics and population distributions of conformational states; and AI (particularly deep learning) helps alleviate major computational bottlenecks of sampling high‐dimensional spaces, enabling more efficient exploration of the conformational landscape [8]. As detailed in subsequent sections, this integrated approach is improving the ability to model protein conformational ensembles (a thermodynamic distribution of discrete states) and time‐correlated dynamics (the kinetic transitions between these states), bridging the gap between microscopic conformational states (captured by simulations) and macroscopic biological function (observed in experiments) (Figure 2).

In this review, we focus on the tripartite synergy of experiments, physics, and AI—with physics as the “integrative glue”—and discuss implications for applications to biological problems. In doing so, we adopt a computational biophysics‐centric perspective to address the following themes: 1) Core principles, strengths, and recent advances in experimental, physics‐based (force field‐driven), and deep learning approaches for investigating protein conformation and dynamics—with a focus on their complementary roles in computational modeling. 2) Strategies and outcomes of integrating these three paradigms via integrative biology frameworks, emphasizing how computational methods synthesize heterogeneous data into predictive models. 3) Finally, we summarize the current computational challenges in integrative modeling and simulation (e.g., force field accuracy, sampling efficiency) and discuss prospects for advancing the computational study of protein dynamics.

## 2 Key, Selected Methods for Investigating Protein Conformations and Dynamics: Strengths, Limitations, and Complementarity

Over the past few decades, experimental techniques, physics‐based simulations, and AI approaches have undergone substantial technological maturation. Experimentally, structural biology has progressed from often studying smaller, monomeric proteins to the high‐resolution characterization of complex, dynamic macromolecular assemblies and their interactions within native cellular environments [16, 17]. More recently, emerging methods have begun to overcome traditional ensemble‐averaging limitations, enabling time‐resolved dynamics at the single‐molecule level and direct reconstruction of conformational ensembles and associated kinetic properties [18].

In parallel, advances in hardware, software, and algorithmic efficiency have dramatically extended the spatiotemporal reach of physics‐based simulations, now permitting microsecond‐to‐millisecond sampling at the all‐atom level and multiscale modeling of large proteins and complex biological processes [19, 20]. Meanwhile, AI‐driven computational methods have achieved transformative breakthroughs over the past decade, with accurate prediction of protein structures now a cornerstone of AI‐assisted biological research. Emerging generative AI approaches enable the modeling of protein systems spanning both thermodynamic free energy landscapes and kinetic time‐correlated dynamics [21, 22].

### 2.1 Experimental Techniques for Determining Protein Conformations and Conformational Ensembles

Experimental techniques are essential to studying protein conformational dynamics, providing complementary structural information across resolutions and timescales. High‐resolution methods, including X‐ray crystallography, cryo‐EM, and NMR spectroscopy, enable atomic‐resolution structure determination. X‐ray crystallography and cryo‐EM primarily target ground‐state conformations but can resolve multiple functional states in heterogeneous datasets, indirectly informing conformational transitions [16, 18]. While these methods excel at capturing static structures, they are generally less effective for directly resolving atomic‐scale temporal evolution. NMR spectroscopy can probe motions across broad timescales, from picoseconds to seconds (e.g., side‐chain rotations to large conformational exchanges) at high spatial resolution, and quantifies exchange rate constants [23]. Complementary lower‐resolution techniques offer valuable structural and dynamic insights. Small‐angle X‐ray/neutron scattering (SAXS/SANS) probes the radius of gyration (*R*_g_), maximum intramolecular distance, and low‐resolution molecular envelopes. SAXS/SANS is well suited for monitoring solution‐state dynamic equilibria, making it suitable for oligomerization and large‐amplitude changes in intrinsically disordered proteins (IDPs) [24, 25, 26]. Atomic force microscopy (AFM) can probe surface topography, and cross‐linking mass spectrometry (XL‐MS) constrains intra‐ and intermolecular distances between specific residues [27]. Hydrogen‐deuterium exchange mass spectrometry (HDX‐MS) tracks backbone amide exchange rates, revealing residue‐level solvent accessibility, hydrogen bonding, secondary structure stability, conformational rearrangements, and binding interfaces [28].

Besides the aforementioned label‐free techniques, several methods rely on covalent attachment of probes to specific protein sites. These approaches yield sparse but highly informative structural and dynamic information, such as inter‐residue distance distributions, enabling estimation of conformational populations and insights into state‐transition mechanisms. Prominent examples include double electron–electron resonance (DEER) [29] spectroscopy and Förster resonance energy transfer (FRET) [30]. DEER measures distance distributions between spin labels via electron–electron dipolar coupling, while FRET quantifies distance‐dependent energy transfer between fluorophores, with time‐resolved and single‐molecule variants (smFRET) further resolving dynamic fluctuations [31]. However, these probe‐based methods have notable limitations: the relatively bulky probes and their flexible linkers can perturb local conformation and function, and the measured distances reflect probe–probe rather than true residue–residue separations.

Experimental studies of protein dynamics face inherent limitations. Most techniques primarily capture stable ground states, metastable intermediates, or equilibrium populations, while high‐energy transition states governing conformational changes remain challenging to characterize directly [1]. Additionally, many experimental observables represent ensemble‐averaged properties rather than individual states due to fundamental and technical constraints.

### 2.2 Physics‐Based Simulation Techniques for Protein Conformational Dynamics

#### 2.2.1 Simulation Methods: Molecular Dynamics and Monte Carlo

Unlike experiments, which directly observe structural data, physics‐based simulations infer conformational dynamics using physically motivated force fields. These methods generate atomistic trajectories that connect microscopic conformational fluctuations with macroscopic biological function [15, 32]. By sampling the Boltzmann distribution under defined environmental conditions, simulations provide atomistic access to structural, thermodynamic, and kinetic properties at resolutions difficult to resolve experimentally [33, 34].

Advanced sampling techniques capture high‐dimensional thermodynamic and kinetic data at atomic resolution with finer structural and temporal resolution than current experiments. Moreover, bridging techniques (e.g., forward models in integrative structural biology that predict experimental observables from proposed structural ensembles, see Section 3.2.1 for details) enable rigorous validation against experimental observables [35].

Below, we focus on commonly used physics‐based methods: Molecular Dynamics (MD) [36], Monte Carlo (MC) simulations [37, 38], and enhanced sampling techniques for overcoming sampling bottlenecks.

MD simulates protein motion by numerically integrating Newton's equations of motion, capturing both local fluctuations and large‐scale conformational transitions. MC instead generates stochastic conformational moves accepted according to statistical mechanical criteria, emphasizing efficient exploration of equilibrium ensembles [39, 40]. These approaches have complementary strengths. MD explicitly resolves temporal evolution and is therefore especially useful for mechanistic studies of dynamical processes. MC, by avoiding explicit force integration, often explores conformational space more efficiently and can more readily cross energy barriers, though it generally lacks direct kinetic interpretability.

#### 2.2.2 Addressing Sampling Challenges

The high dimensionality of protein conformational space and large free energy barriers often trap standard MD/MC simulations in local free energy minima, limiting sampling of biologically relevant transient states. To address this, two key strategies are widely used (alongside hardware acceleration of mainstream packages like GROMACS [41, 42], OpenMM [43], LAMMPS [44], AMBER [45], CHARMM [46], NAMD [47]): coarse‐grained (CG) modeling and enhanced sampling [48].

##### 2.2.2.1 Coarse‐Grained Modeling

Coarse‐grained simulations follow a modeling framework in which groups of atoms or molecules that exhibit similar chemical character or are spatially proximal are represented collectively by a single interaction site, often termed a “bead”. This reduction in the number of degrees of freedom substantially lowers computational cost. The effective interaction potentials employed in CG models are typically parameterized either through direct calibration against all‐atom force fields or by matching experimentally observable properties of the target system (or both) [49]. The widely used Martini model, for example, typically consolidates three to five side‐chain heavy atoms of a residue into one bead or treats clusters of water molecules as a unified solvent particle [50, 51, 52].

As CG models often do not capture the subtle balance of atomic interactions that stabilize protein structures, folded domains are generally unstable in these models. Thus, CG are often used together with structure‐based approaches (e.g., elastic network model (ENM) and Gō‐like models) that are often built on the protein's native structure to capture conformational dynamics [53].

Relative to all‐atom representations, CG approaches may provide orders‐of‐magnitude gains in computational efficiency, thereby granting access to system sizes and simulation timescales that remain computationally challenging with fully atomistic descriptions. However, this enhanced spatiotemporal reach comes at the expense of atomic‐level structural and energetic resolution.

##### 2.2.2.2 Enhanced Sampling Techniques

Enhanced sampling is an additional strategy to boost conformational exploration efficiency. Many of these methods rely on dimensionality reduction via collective variables (CVs)—descriptors also sometimes called reaction coordinates or order parameters, depending on study context and system properties. Appropriate CV selection can substantially improve sampling efficiency by focusing simulations on the most relevant regions of conformational space; poor choices, by contrast, may lead to incomplete or misleading sampling or converging to unphysical states. Though trajectories are biased, proper post‐simulation reweighting procedures can, in some cases, recover unbiased equilibrium distributions and kinetic information [54].

The choice of which enhanced sampling protocol to use can be informed using structural and mechanistic insight into the system and process at hand. Given prior knowledge of the system—such as key residues involved in a transition, known conformational states, or mechanistic hypotheses—or informed by expert intuition, researchers can select CV‐based methods that are relevant to the process of interest, ensuring targeted and efficient exploration. Representative CV‐based methods include the classical umbrella sampling (applies harmonic bias potentials to sample overlapping windows) [55], metadynamics (iteratively deposits repulsive Gaussian bias potentials along predefined CV to push the system out of local free energy minima) [56, 57] and its derivatives such as OPES [58, 59, 60], integrated tempering sampling [61], adaptive biasing force (ABF) [62], and unified methods (e.g., metadynamics‐xABF [63], oneOPES [59] and OPES with expansion CVs [64]). Recent advances include the machine learning (ML)‐assisted identification of optimal CVs and adjustment of bias potentials [65], which are elaborated in “*Section* *5.2* *, AI for Physics: Advancing MD Simulations through AI”*.

For systems with poorly characterized slow degrees of freedom and insufficient prior knowledge to define meaningful CVs, “CV‐free” enhanced sampling methods provide an alternative. Sometimes these approaches directly or indirectly use the potential energy as a CV. These approaches including REMD (both temperature‐based or Hamiltonian‐based variants) [66], accelerated MD (aMD) and its Gaussian variant GaMD (the boost potential follows Gaussian distribution), which use global properties (e.g., potential energy) to drive global conformational exploration, facilitating exploration across large entropic barriers and access hidden/orthogonal degrees of freedom overlooked by CV‐based approaches [67]. Such CV‐free methods do not require detailed prior knowledge of the protein conformational processes involved, making them suitable for biomolecular systems that lack adequate prior information and for which it is difficult to define effective CVs capable of achieving significant acceleration. In addition to these biased approaches, a complementary set of methods relies on unbiased simulations without modifying the energy landscape, such as transition path sampling [68], Markov state modeling (MSM) [69], Milestoning [70, 71], and weighted ensemble [72].

Enhanced sampling is an active area of methodological development. Given the scope of this review, we highlight only key conceptual advances and refer readers to recent comprehensive reviews for detailed methodological discussions [54, 73, 74].

Sampling methods for MD simulations have been widely used across numerous research domains, including studies of protein folding, protein‐ligand binding, and allostery. Nevertheless, for large protein systems undergoing complex conformational transitions, sufficient sampling remains a formidable challenge. The high adaptability and flexibility of physics‐based sampling approaches make them useful tools for integration with complementary techniques to investigate dynamical processes.

### 2.3 AI‐Driven Approaches for Protein Conformational Dynamics

#### 2.3.1 Evolution of Deep Learning for Protein Structure Prediction

Unlike traditional physics‐based methods, AI has enabled increasingly efficient analysis, prediction, and generation of protein conformations and ensembles by learning sequence–structure–dynamics relationships from large experimental or simulation datasets; recent advances in hardware (e.g., high‐performance GPUs and specialized AI accelerators like Tensor Processing Units, TPUs) and algorithms (e.g., variational autoencoders (VAEs), transformers, graph neural networks (GNNs, that model molecular topological features), and generative adversarial networks (GANs)) have achieved promising accuracy in key tasks including protein structure prediction, protein–protein/ligand complex modeling, conformational ensemble generation, and de novo design (e.g., RFdiffusion [75], ProteinMPNN [76], and La‐Proteina [77], Odesign [78], etc.). These breakthroughs have gradually shifted research toward integrated multi‐model frameworks that help overcome intrinsic drawbacks of classical experimental and computational tools, potentially pushing forward protein biophysics research by facilitating rapid, large‐scale exploration of proteome‐wide conformational landscapes [79].

Deep learning forms the foundation of AI's major impact on structural biology. AlphaFold2 uses attention‐based architecture and iterative optimization achieved near‐experimental accuracy in single‐protein structure prediction, greatly advancing large‐scale proteome analysis [80]. AlphaFold3 [81], RFdiffusion3 [82], and related methods have further expanded toward broader biomolecular modeling (integrating proteins, DNA, RNA, ligands, ions, and modified residues) via diffusion models that directly generate atomic‐level coordinates.

#### 2.3.2 Generative AI for Conformational Ensembles and Trajectories

Given the rapidly expanding range of generative AI techniques optimized for conformational ensemble prediction and trajectory simulation, we focus on representative methodologies to ensure conciseness—acknowledging that numerous other valuable approaches exist but cannot be fully covered here [83]. Modern generative AI has substantially expanded the scope of protein conformational ensemble prediction by integrating explicit biophysical dynamics into prediction and design pipelines. These approaches follow distinct methodological paradigms defined by their core objectives: some models are explicitly designed to sample from predefined likelihood distributions, while others prioritize generating conformational diversity with less strict likelihood constraints.

For example, diffusion models generate structures via two processes: a forward process that progressively adds Gaussian noise to native protein conformations, and a reverse iterative denoising process that recovers physically plausible structures from corrupted random states. The denoising process is conceptually similar to protein folding dynamics. Representative architectures include RFdiffusion and AlphaFold3. Flow‐based models (including normalizing flows and flow‐matching [84]) offer several advantages in generating protein ensembles via invertible neural networks for modeling continuous conformational distributions. Unlike the iterative denoising in diffusion models, they establish direct invertible mappings between (high‐dimensional) protein conformations and simpler latent spaces, enabling efficient sampling of novel conformations and exact likelihood estimation—useful for estimating ensemble likelihoods. They are useful for continuous conformational distributions, with improved ensemble sampling and diversification, as exemplified by AlphaFlow and ESMFlow, which use flow‐matching to extend static predictors (AlphaFold and ESMFold [85]) for rapid sampling of conformational ensembles.

Transformers and large language models (LLMs) are emerging as promising tools for ensemble and trajectory generation. By tokenizing continuous atomic coordinates into discrete sequences, these models can represent and predict MD trajectories, though current applications remain limited to smaller biomolecular systems. A key advantage of this approach is its flexibility, allowing researchers to either prioritize conformational diversity or employ likelihood‐guided sampling. Their ability to capture long‐range structural correlations suggests broader future applicability even with current limitations in scalability [86].

#### 2.3.3 Limitations of Current AI Approaches for Modeling Protein Dynamics

Current AI‐based methods for modeling protein dynamics still face two major challenges: data scarcity and limited interpretability. On the one hand, insufficient data restricts the generalizability of AI models and impairs their capacity to capture the diversity of protein dynamic behaviors. On the other hand, a lack of interpretability hinders researchers from extracting mechanistic insights from model outputs and makes it difficult to quantify confidence in the predictions. To overcome these limitations, two parallel advances are required: first, the optimization of experimental and computational simulation techniques to generate more diverse, high‐quality datasets of protein dynamics; second, the development of physics‐informed AI architectures that inherently integrate the physical principles underlying protein behavior.

Although static protein structural databases are well established, such as the Protein Data Bank (PDB) [87, 88], and may implicitly encode clues about dynamic characteristics, experimentally determined dynamic protein ensembles remain scarce, (e.g., Small Angle Scattering Biological Data Bank, SASBDB [89], and PDB‐IHM [90], a dedicated archive for conformational models and ensemble structures, along with the associated primary experimental data, obtained through integrative or hybrid modeling approaches). Public MD trajectory repositories (e.g., MDDB [91], MoDEL [92], Dynameomics [93], ATLAS [94], mdCATH [95], DynaRepo [96], ProteinConformers [97]) predominantly feature sub‐microsecond simulations, with only few individual simulations (e.g., from D. E. Shaw Research) [98] reaching millisecond timescales. This data insufficiency stems from the computational challenge of accessing functionally relevant microsecond‐to‐millisecond transitions, restricting long‐timescale simulations mostly to small peptides or single‐domain proteins. A broad inventory of protein conformation databases, covering both static single‐structure and dynamic/ensemble‐based resources, is provided in Table 1. Despite recent progress, the volume and diversity of such data remain inadequate for training generalizable AI models of proteome‐wide protein dynamics.

**TABLE 1:** Protein Conformation Databases.

Database	Systems	Source	Time scale per system
Single‐structure Databases			
PDB [87]	Monomers & Complexes	X‐ray, NMR, cryo‐EM	—
HSP [88]	Monomers	PDB	—
AFDB [99]	Monomers	AlphaFold Prediction	—
AFESM [100]	Monomers	AFDB and ESM Metagenomic Atlas	—
Dynamics/Ensemble Datasets			
SASBDB [89]	Monomers & Complexes	SAXS & SANS	Ensemble
PED [101]	Monomers & Complexes	SAXS & NMR & FRET	Ensemble
MoDEL [92]	Monomers	MD	0.001–1 µs
Dynameomics [93]	Monomers	MD	∼0.1 µs
ATALS [94]	Monomers	MD	∼0.1 µs
mdCATH [95]	Domains	MD	<0.5 µs
DynaRepo [96]	Monomers & Complexes	MD	∼0.5 µs
DynamicPDB [102]	Monomers	MD	∼1.0 µs
ProteinConformers [97]	Monomers	MD (very short)	∼0.1–0.4 ns
D. E. Shaw MD [98]	Miniproteins (14)	MD (long)	0.1–1 ms
Integrative Database			
PDB‐IHM [90]	Large Complexes	Hybrid/integrative modeling	Ensemble

## 3 Integration of Experiment and Physics: Extracting Insights via Physics‐Based Methods

**FIGURE 3:**
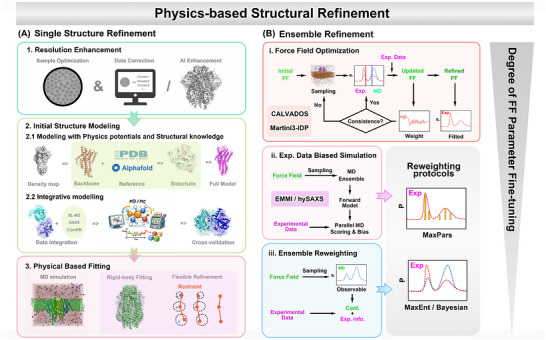
Workflows for physics‐based refinement of single protein conformations and conformational ensembles. (A) An example of the workflow for deriving stable single‐conformation models from experimental density maps. Resolution may first be improved through optimization of the experiments, computational data correction, and AI‐driven enhancement techniques. A reliable initial atomic model is then generated by integrating physicochemical principles with optimal density fitting, drawing on homology or machine‐learning predictions, template‐based tracing, or integrative restraint‐guided approaches (e.g., incorporating XL‐MS, HDX‐MS, and NMR data). The final step uses force field‐guided fitting, including rigid‐body alignment (Chimera, Rosetta) and flexible refinement (MDFF, ISOLDE) to produce a physically consistent, high‐resolution structure. (B) Strategies for extracting conformational ensemble information guided by experimental data. Contemporary approaches primarily encompass three complementary paradigms: (i) iterative optimization of the underlying force field to improve agreement with experimental observables; (ii) experiment‐biased molecular dynamics simulations in which experimental restraints are introduced as biasing potentials to direct ensemble sampling; and (iii) reweighting of pre‐generated conformational samples to maximize consistency with experimental density and dynamic data (e.g., using MaxPars, MaxEnt, or Bayesian methods).

Experimental techniques and physics‐based simulations have complementary strengths and limitations: experiments provide direct observations of protein structure and dynamics (e.g., ensemble‐averaged distances), but may lack atomic resolution, temporal detail, or access to transient intermediates; simulations offer atomic‐scale trajectories, thermodynamic (e.g., free energies) and kinetic information but are constrained by sampling bottlenecks and force‐field inaccuracies. Integrating these approaches—using experimental data to guide/constrain/validate simulation models and simulations to interpret experimental observables via physical mechanisms—overcomes individual limitations, enabling more detailed biophysical interpretation into conformational ensembles. This synergy facilitates the interpretation of experimental observations using quantitative physical models for comprehensive characterization of protein structure, dynamics, and function. Below, we elaborate on the integration of physics and experiments from the perspective of two central tasks in structural biology: single‐conformation refinement and ensemble refinement (Figure 3).

### 3.1 Physics‐Based Refinement of Single Protein Conformation

Single‐structure protein refinement generates physically plausible 3D structures consistent with experimental data and biophysical principles. Unlike data‐driven methods that prioritize experimental fitting over structural realism, physics‐based approaches integrate force fields and molecular mechanics into workflows, ensuring consistency with experimental measurement and physical constraints [103]. The process has three core steps: experimental data resolution enhancement, initial structural modeling, and force field‐guided fitting.

Resolution enhancement and initial modeling address the challenge of extracting high‐resolution details from low‐resolution experimental data. Two strategies are used: (1) combining experimental restraints with physics‐derived energy functions; (2) applying knowledge‐driven potentials from high‐resolution structure databases. For large macromolecular assemblies, IMP (Integrative Modeling Platform) [104] integrates heterogeneous data via a four‐stage protocol (input collation, restraint translation, model sampling, validation), enabling modeling of intractable systems (e.g., nuclear pore complexes) and supporting PDB‐Dev deposition [105] with spatiotemporal extensions.

Initial models provide initial residue‐level coordinates at nominal resolution, but flexible regions (e.g., side chains, coordinated ions, and lipids) are often poorly defined due to conformational averaging, requiring further force field‐guided refinement. Conventional rigid‐body fitting and real‐space refinement (Chimera [106], Rosetta [107], PHENIX [108]) can improve the model at moderate‐to‐high resolutions, MD‐based refinement better explores local conformational space and resolves mechanistic details, but is computationally costly [109]. Methods like MDFF integrate electron‐density‐derived potentials into standard MD for local conformational adjustments [110, 111], while REFMAC5 [112] and ISOLDE [113] adopt simplified force‑field terms to achieve faster optimization.

Physics‐based methods for refining individual protein structures are relatively mature, whereas recent advances in structure optimization have primarily focused on AI‐driven approaches, which will be discussed further in Section 4.

### 3.2 Physics‐Based Extraction of Protein Conformational Ensembles From Experimental Data

#### 3.2.1 Forward Models: Bridging Simulations and Experiments

Most experimental measurements represent ensemble averages of observables over a certain timescale and typically a large number of molecules. From the perspective of single‐structure optimization, the conformational heterogeneity embedded in such data constitutes “noise,” posing significant challenges to accurate structure determination. However, this heterogeneity inherently contains information about protein dynamics [114, 115]. To extract the information contained within experimental data, it is therefore useful to construct and refine ensembles from the data itself. Such ensemble refinement is, however, more challenging than single‐structure refinement. One effective strategy is thus to integrate experimental data with MD simulations, leveraging the latter to sample the dynamic information implicit in the former [114].

Prior to integrating experimental data with MD simulations, a mapping relationship is typically established to project molecular properties from both sources onto a common set of observables, enabling direct comparison and subsequent optimization of MD trajectories. Owing to the much higher dimensionality of MD atomic coordinates, a so‐called “forward model” is typically used to bridge 3D molecular structures to experimental observables [116]. Forward models refer to mathematical frameworks that, starting from a known molecular structure or conformational ensemble (usually obtained from the PDB or MD trajectories), compute the expected experimental observable under defined measurement conditions. These models employ physical and mathematical formalisms to compute corresponding experimental measurements, such as those from SAXS, cryo‐EM, DEER, FRET, and NMR paramagnetic relaxation enhancement (PRE), nuclear Overhauser effect (NOE), and residual dipolar coupling (RDC). Classic forward models include the Debye formula for SAXS intensity calculation from atomic structures [114, 117, 118], and rotamer library‐based approaches (e.g., DEER‐PREdict and FRETpredict) [119, 120, 121]. The latter approaches are used for predicting DEER distance, PRE rates or FRET efficiencies distributions from MD ensembles, which help reduce systematic errors arising from the size of the probes (discussed in 2.1). To speed up calculations, it is also possible to use CG representations, such as one‐bead‐per‐residue models with on‐the‐fly solvation layer corrections for efficient SAXS/SANS prediction in MD simulations. Conversely, at an increased computational cost it is also possible to incorporate solvent effects more explicitly. These methods can be used with the Metainference‐based frameworks that account for errors in forward models during ensemble refinement. Complementary approaches further enable joint refinement of conformational ensembles and forward model parameters in a self‐consistent manner [122].

#### 3.2.2 Statistical Frameworks for Ensemble Refinement

Forward models facilitate direct comparison between simulated and experimental structures, allowing iterative strategies to minimize discrepancies between simulated conformational ensembles and experimental averages. These strategies fall into three broad categories: maximum parsimony, maximum entropy, and Bayesian inference, though the latter two are closely related [118, 123].

The maximum parsimony method minimizes deviations from the prior simulated ensemble, reweighting structures only to the extent strictly necessary for experimental agreement [124, 125]. In contrast, the maximum entropy approach selects conformational ensembles that introduce the least additional information beyond experimental restraints, yielding distributions consistent with observations while minimizing bias. Bayesian inference treats ensemble weights as posterior probabilities, combining likelihood terms derived from simulated data with prior assumptions (typically from initial MD distributions), while quantifying uncertainty in the resulting ensembles [123, 126].

The principle of maximum entropy can be formulated as providing the least‐biased probability distribution and is thus closely related to Bayesian methods. Consequently, integrative structural modeling methods often employ Bayesian frameworks, such as BICePs [127, 128], BioEn [123, 129], ISD [130, 131], MELD, and BELT [132], Bayesian Weighting [133], etc. Advances in Metainference and Bayesian reweighting techniques have further supported the widespread use of Bayesian frameworks, enabling uncertainty quantification and mitigation of overfitting when handling multi‐source, heterogeneous, and noisy experimental data.

#### 3.2.3 Technical Frameworks for Ensemble Refinement

In the process of refining simulations with experimental data to construct conformational ensembles, technical frameworks can roughly be classified—based on the extent of force‐field modification from extensive to minimal—as follows: force‐field refinement, experimental data‐biasing, and post‐simulation reweighting. While the former approach attempts to provide transferable improvements to the force field, the two latter approaches derive system and data‐specific force field corrections.

##### 3.2.3.1 Force‐Field Refinement

For a given structure, constructing conformational ensembles through MD sampling is among the most intuitive and direct strategies. This is because the force field encodes the underlying potential energy surface governing protein folding and dynamics. Accordingly, the fidelity of the derived conformational ensembles, as well as their agreement with experimental observations, depends directly on the employed force field parameters. After decades of refinement, contemporary all‐atom force fields achieve good overall accuracy. However, systematic deviations are often observed when modeling highly flexible proteins, subtle local allosteric transitions in large biomolecular systems, and in longer timescale dynamic processes—limitations might be inherent to their fundamental parameterization schemes. General force fields are typically parameterized by fitting against experimental datasets and high‐level quantum mechanical (QM) calculations of small molecules or peptides, followed by validation on larger systems [134].

Advances in quantum chemistry and high‐throughput data generation have accelerated the development of molecular force fields, enabling improved modeling of protein dynamics and conformational heterogeneity. For instance, widely adopted all‐atom general force fields (such as from the CHARMM and Amber families) have undergone continuous refinement release, with extensions to non‐protein parameters as well as targeted improvements for IDPs through reweighting based on QM calculations and experimental data [135, 136, 137, 138]. Open‐source initiatives, such as the Open Force Field (OpenFF) [139], have accelerated progress by employing standardized training datasets and automated parameter fitting workflows [140]. These, in turn, build on Bayesian formulations of how to learn force field parameters from experiments [141, 142].

CG force field development has similarly advanced rapidly in recent years. The 2021 release of Martini 3 comprehensively reparametrized the protein force field by introducing finer bead types, though initial coverage of organic small molecules remained limited [50]. AutoMartini3 effectively addressed this gap by enabling high‐throughput parameterization through automated mapping and bonded parameter generation, supporting larger molecules and broader chemical space [143, 144]. To mitigate dynamical distortions in IDP simulations, early efforts included scaling adjustments on bead interactions; for example, a slight enhancement of protein–water Lennard–Jones interactions or minor reduction of protein‐protein interactions has been shown to improve agreement with SAXS data for IDPs and multi‐domain proteins while enhancing consistency with PRE measurements [145, 146]. Martini and related “medium resolution” CG models are complemented by even coarser representations such as the hydrophobic‐polar‐solvated (HPS) model family. For example, the CALVADOS protein force field represents each amino acid by a single bead. It was originally designed for simulating IDPs/IDRs and associated phenomena, such as their self‐assembly and phase separation (PS) [147]. The 2023 CALVADOS 2 version [148], optimized for pure IDPs, relatively accurately reproduces single‐chain conformational expansion (e.g., *R_g_*), phase separation behavior, and solution‐phase experimental observables (e.g., SAXS profiles), incorporating a simplified model for salt‐screening effects. Later iterations, such as CALVADOS 3 and its extensions [149, 150], extend applicability to multi‐domain proteins while preserving IDP accuracy and improving descriptions of folded‐domain–disordered‐region interactions, often in conjunction with ENMs to maintain folded‐domain integrity. Martini3‐IDP has been introduced as a dedicated extension of Martini 3, ameliorating some of the issues related to the description of disordered proteins and protein‐protein interactions present in the original Martini3 model [148, 151]. Like earlier related approaches [152, 153, 154], this model enhances IDP conformational entropy, accuracy in fuzzy complexes, and phase separation simulations, while remaining suitable for complex environments, such as membrane‐associated IDP systems.

##### 3.2.3.2 Experimental Data‐Biasing: Guiding Simulations with Restraints

Experimental data‐biasing is an intermediate modification strategy that avoids direct force field alterations, instead applying real‐time restraints during MD simulations to steer systems toward conformations consistent with experimental observables [155]. It leverages forward models to compute discrepancies between simulated and experimental values, then uses biasing potentials (e.g., harmonic or flat‐bottom wall restraints) to minimize these differences [156, 157, 158]. Conceptually, the approach preserves the inherent energy landscape described by the force field, while targeting experimentally relevant regions of conformational space. Common implementations include restraining NMR NOE‐derived distances, orientations from RDCs, or SAXS intensity profiles. A major strength of this strategy lies in its high flexibility: biasing potentials can be dynamically adjusted to focus on specific observables or gradually relaxed to allow exploration of alternative conformational states. Nevertheless, this method may suffer from tuning of biasing potential (too weak potentials fail to guide effectively; too strong potentials may trap systems in non‐physical conformations).

The maximum entropy principle introduced above applies well to this scenario. By incorporating an experimental constraint bias into a modified energy function, the Kullback–Leibler divergence between the resulting updated distribution and the reference (original) force‐field distribution can be quantified, enabling distribution optimization under the maximum entropy principle.

A more direct alternative imposes penalty terms to quantify discrepancies between experimental observables and forward model predictions from instantaneous simulation conformations. Such penalties are added as supplementary potential energy terms, increasing the effective energy of conformations with large experimental deviations and thereby suppress their sampling probability. Given the inherent uncertainties associated with experimental measurements and forward model approximations, the conventional maximum entropy framework requires tailored modifications to take these errors into account [114, 123, 156]. Implementations include Experiment Directed Simulation (EDS) [159, 160] and Experiment Directed Metadynamics (EDM) [161], which apply adaptive linear biases to selected CVs during MD simulations. These methods iteratively refine bias parameters via optimization algorithms to align simulated FES or forward‐modeled observables with experimental target averages. Software platforms like PLUMED [162] (specialized in CV definition and bias potential application) are particularly amenable to these integrative simulation workflows. Notably, PLUMED's Integrative Structural and Dynamical Biology (ISDB) module provides pre‐implemented restraint functions for diverse experimental data types (SAXS, cryo‐EM densities, NOE distances, FRET, etc.), facilitating the integration as biasing terms in MD simulations [163].

Bayesian approaches have advanced rapidly for experiment‐guided ensemble refinement, providing a statistically grounded framework to link prior physical knowledge (from simulations) with experimental observable‐informed posterior distributions, formally expressed as:

$$\underset{posterior}{\underset{︸}{P(X,\sigma |O^{\exp})}}\propto \underset{priors}{\underset{︸}{P(X)•P(\sigma )}}•\underset{likehood}{\underset{︸}{P(O^{\exp}|X,\sigma )}}$$

here, the experimental observables (*O* ^exp ^) constitute known data; thus, the practical objective is to refine the simulated distribution by fitting it to the experimental observations via the likelihood term. In algorithmic implementation, deriving functional forms for these three components typically involves four key steps: (1) construction of a forward model, (2) development of a noise model, (3) specification of prior distributions, and (4) sampling via MD or MC methods. For simpler cases, a forward model alone may suffice to define a basic likelihood; however, real‐world data often require careful accounting for measurement uncertainties to avoid overfitting. This is typically achieved by introducing an error parameter, σ, to construct a Bayesian likelihood function, thereby extending the forward model into a probabilistic noise model. Within this framework, the structural prior is derived from the Boltzmann distribution encoded in the force field itself, while the prior on the experimental noise is informed by the statistical properties of the associated experimental uncertainties. Maximizing the posterior steers simulations toward conformations reconciling physical principles and experimental constraints. To align experimental data heterogeneity with protein ensemble properties, Metainference frameworks have been developed based on standard Bayesian inference [114, 164]. These use multiple parallel MD replicas to generate conformations and construct forward models by comparing experiments to “pseudo‐ensembles” of cross‐replica combinations, improving fits to complex, heterogeneous datasets [165].

Leveraging these foundational frameworks, a suite of specialized tools has been developed to tackle the distinct challenges associated with integrating specific experimental data modalities. hySAXS employs hybrid‐resolution structural representations to strike a balance between computational efficiency and structural accuracy in SAXS profile fitting [166, 167]. Expanded to SANS as hySAS [118], this tool integrates ultra‐CG and real‐time solvation correction schemes, thereby improving scattering profile calculations for complex systems involving contrast variation or deuteration modeling. Integrating Gaussian mixture models (GMMs) into meta‐inference decomposes cryo‐EM density maps, enabling atomic position identification in low‐resolution maps. This led to EMMI (Maximum Entropy Meta‐Inference) [158, 168] and enhanced MEMMI [169, 170]—strengthened by PB‐Metadynamics for rare state sampling—suitable for medium‐sized soluble proteins and membrane protein domains. Experimental data‐biasing bridges force field‐based MD simulations and experiments, with maximum entropy/Bayesian frameworks and tools enabling multi‐modal data integration. These advancements expand MD simulation scope, facilitating previously inaccessible studies of biomolecular dynamics and function. However, it should be noted that, similar to force‐field optimization approaches, this kind of distribution‐improving strategies require substantial computational resources to achieve converged sampling. Moreover, because most applied bias potentials act as forces during the simulation iterations, large instantaneous discrepancies between the initial input structure and experimental data may cause simulation instability and crashes, necessitating expert‐level parameter fine‐tuning and resulting in a relatively steep learning curve for practical applications.

##### 3.2.3.3 Post‐Simulation Reweighting

Post‐simulation reweighting represents an alternative modification strategy. This method first performs exhaustive MD sampling to generate comprehensive conformational pools, followed by statistical reweighting to select optimal conformational subsets and assign corresponding weights that best reproduce experimental observations. Subsequent reweighting recalibrates the original Boltzmann probability distributions of simulated conformations, prioritizing structural populations whose forward‐model‐derived observables achieve global consistency with experimental measurements at the ensemble level.

One practical advantage of post‐simulation reweighting is its computational efficiency and simplicity: unbiased simulations can be run once and reweighted multiple times to integrate different experimental datasets or test alternative forward models, eliminating the need for repeated simulations [171]. Additionally, this method lowers the risk of perturbing the underlying simulation dynamics, as the raw MD sampling is unperturbed by experimental restraints. However, its effectiveness is contingent on the unbiased simulation sufficiently sampling all experimentally relevant conformational states—if key states are not populated in the initial trajectory, reweighting cannot recover them.

Reweighting methodologies frequently rely on Bayesian formulations [123, 172, 173, 174], or the related MaxEnt principle [175, 176, 177, 178, 179, 180, 181]. Such a choice minimizes the introduction of additional information beyond what is strictly required by the data, thereby yielding the least biased ensemble consistent with observations. To balance fidelity to the original simulation, the optimization typically incorporates a regularization term in the form of relative entropy between the reweighted distribution and the prior (uniform or simulation‐derived) weights. Experimental constraints are encoded as average observables, and the resulting constrained optimization problem is solved iteratively using Lagrange multipliers or equivalent numerical techniques.

Another set of methods instead employs the MaxPars principle. Here, the guiding philosophy is to achieve agreement with experimental data with the smallest possible set of configurations. This is operationalized by introducing a penalty that discourages the effective population of additional conformations—often through an L1‐like regularization on the weight changes. Consequently, MaxPars tends to produce sparser ensembles with fewer substantially populated states [182]. While this approach is robust to imperfections in the initial MD sampling (e.g., incomplete coverage of conformational space), it can struggle with highly disordered or high‐entropy systems, where enforcing excessive sparsity may lead to inadequate fitting of the data. Conceptually and somewhat simplified, the distinction between MaxEnt and MaxPars can be viewed as differences in what the “simplest” solution is; in MaxEnt it is the broadest possible distribution (for example, closest to the distribution generated by MD), while in MaxPars it is the smallest number of distinct configurations that can explain the data.

These three methods—force‐field refinement, experimental data biasing, and post‐simulation reweighting—are not mutually exclusive and can be integrated to capitalize on their respective strengths and offset individual limitations. Despite these synergistic benefits, there have only been a few such combinations, primarily due to heightened computational complexity (e.g., optimizing expanded parameter sets, ensuring cross‐method compatibility, validating integrated workflows) and the absence of standardized integration protocols, which force researchers to develop custom system‐ or data‐specific pipelines.

## 4 Integrating Experiment and AI: Machine Learning for Conformational Modeling and Ensemble Generation

Dynamic experimental data can also be integrated into AI pipelines, either in training to boost model generalization or as physical constraints to guide sampling toward conformationally relevant solutions aligned with observed physicochemical properties.

For cryo‐EM density maps, deep learning methods can outperform traditional methods in map enhancement and atomic modeling. Early 3D U‐Net tools (e.g., DeepEMhancer) [183] generated LocScale‐comparable maps; subsequent advances like EMReady [184] (3D Swin‐Conv‐UNet) eliminated LocScale dependence by training on noise‐free simulated data and integrating local/global feature capture. Recent Transformer‐based tools (e.g., CryoTEN) [185] improve computational efficiency. Self‐supervised deep learning tools (CryoFM [186], Blush, spIsoNet) further refine maps via denoising, resolution enhancement, and bias mitigation, and are increasingly complementing traditional mathematics‐based approaches as the preferred choice among structural biologists. For atomic model construction medium‐resolution maps, lightweight deep learning tools such as A2‐Net [187], Cryo‐Net, AAnchor [188], DeepTracer [189], CryoSeek [190] enable rapid, physically consistent all‐atom model construction with a relatively low computer cost. Driven by the progress of AlphaFold‐like structure predictors, hybrid modeling tools including ModelAngelo [191, 192], CryoAtom [193], EMProt [194], DeepMainMast [195], EModelX [196], MICA [197] further integrate AI‐predicted structural references to achieve robust modeling of diverse protein systems.

Beyond cryo‐EM, AI has been increasingly integrated with a broad range of biophysical experimental techniques that report on protein dynamics and conformational heterogeneity, including DEER spectroscopy, FRET, SAXS, and HDX‐MS. AI enhances the analytical power of these methods, enabling improved characterization of protein conformations and dynamic transitions. For example, DEERFold modifies the AlphaFold2 architecture to directly incorporate distance distributions derived from DEER spectroscopy, allowing for the guided prediction of multi‐conformational ensembles and the modeling of conformational transitions in membrane transporters [198]. DeepDEER employs convolutional neural networks (CNNs) to process DEER spectroscopy data, which improves the resolution of distance distributions and enables better characterization of protein conformational heterogeneity [199]. decodeSAXS combines a CNN auto‐encoder with SAXS measurements to decode low‐resolution profiles, aiding conformational ensemble reconstruction and bridging SAXS data with structural insights [200]. DeepFRET uses CNNs to analyze single‐molecule FRET data, resolving complex conformational states and quantifying dynamic transitions more accurately than traditional methods [201].

Together, these hybrid AI–experiment frameworks help bridge the gap between static structural determination and dynamic ensemble characterization, translating sparse, low‐resolution experimental readouts into quantitative mechanistic insights into protein conformational landscapes.

## 5 Integrating AI and Physics: Mutual Enhancement for Dynamic Modeling

**FIGURE 4:**
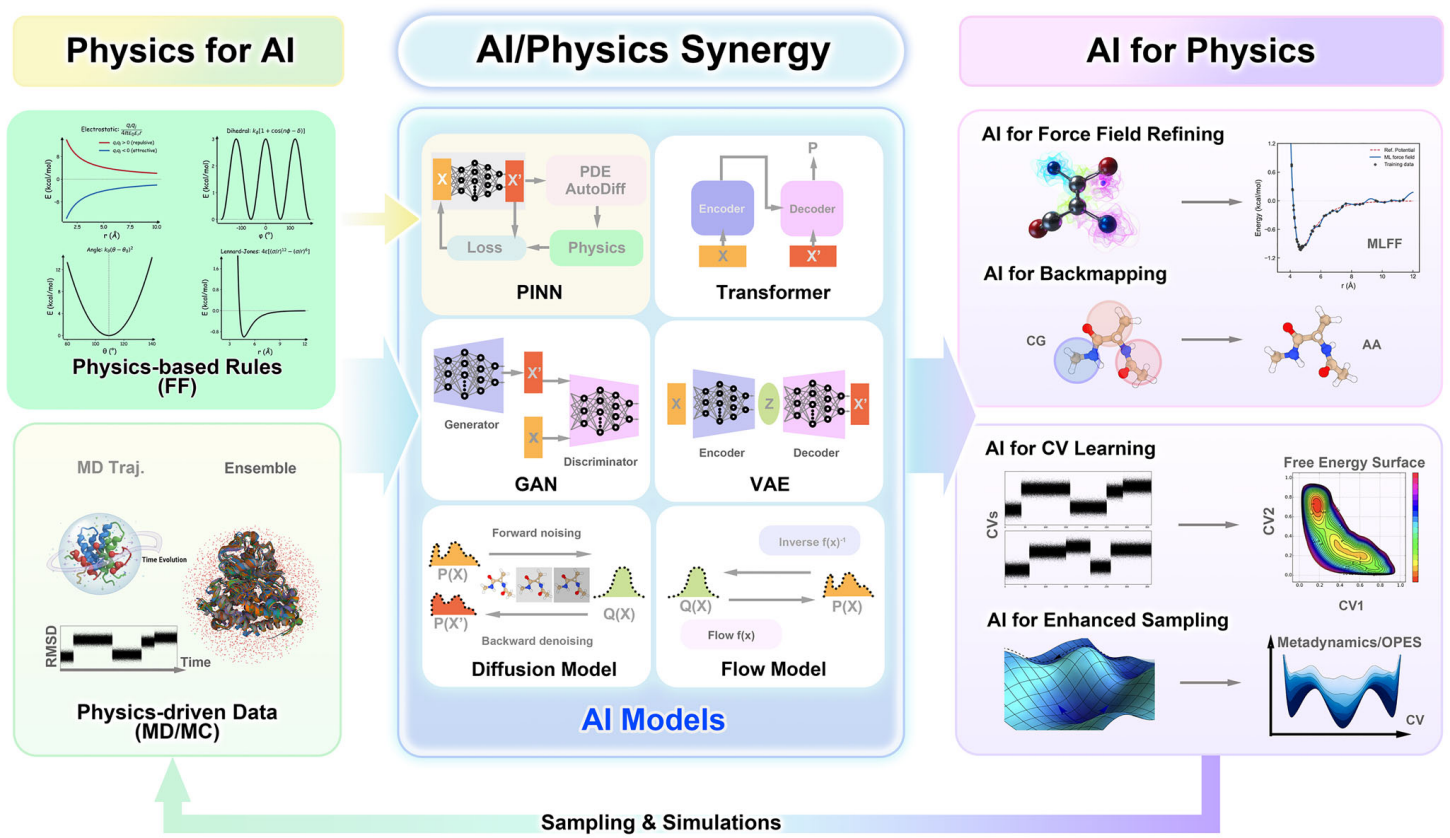
Bidirectional synergy between AI and physics for protein dynamic modeling. (Left) Physics for AI. Physics‐based rules (force fields, FFs) and physics‐driven data (molecular dynamics/Monte Carlo (MD/MC) ensemble sampling and trajectories) provide physical constraints and informative priors for AI model development. This includes generative adversarial networks (GANs), variational autoencoders (VAEs), diffusion/flow models, physics‐informed neural networks (PINNs), and Transformers. By embedding physical priors, the framework ensures that generated protein conformational ensembles and trajectories are more physically plausible and thermodynamically consistent. Concurrently, AI algorithms further optimize outputs via feature selection, free energy analysis, and dimensionality reduction. (Right) AI for Physics. AI augments physics‐based simulations in multiple key ways: it enables the construction of machine‐learned force fields (MLFFs) with enhanced accuracy relative to classical force fields; facilitates backmapping from coarse‐grained (CG) to all‐atom (AA) structural resolutions; automates feature selection and CV identification for MD analyses; and accelerates enhanced sampling of rare dynamical events. The physics‐driven data derived from these AI‐enhanced simulations are then fed back into AI models, closing the AI–physics loop for iterative, integrated modeling.

The integration of AI, particularly deep learning and generative models, with protein biophysics and MD simulations is helping drive the shift from static structural prediction to a deeper understanding of dynamic functional mechanisms [202, 203]. Training on such temporally resolved MD data extends the remarkable capacity of current protein AI tools, which excel at predicting individual structures, to the generation of sequential dynamic trajectories. This approach may partially alleviate the need for stepwise numerical integration (where contemporary simulations typically employ integration timesteps on the order of 1–2 fs, as larger steps risk instability), potentially improving sampling efficiency for certain systems for rare events [21, 22]. Conversely, for MD simulations, AI offers substantial advancements by refining or even describing force fields to achieve greater accuracy [204]. Moreover, AI leverages its data‐analysis capabilities to identify CVs associated with slow dynamics, elucidate rate‐limiting steps in allosteric transitions, and facilitate enhanced sampling strategies grounded in these CVs [205]. Ultimately, the synergy between AI and physics‐based methods represents a novel paradigm that enables both approaches to complement their respective limitations (Figure 4), fostering mutual enhancement and opening new avenues for discovery in protein science [206].

### 5.1 Physics for AI: Enhancing Physical Plausibility of AI‐Generated Structural Dynamics

#### 5.1.1 AI‐Generated Trajectories: Architectures and Advances

In recent years, the rapid advancement of generative AI models (such as diffusion models and flow‐matching frameworks) has helped develop AI methods with the capability to describe some aspects of the dynamic evolution of protein conformations. A prominent application in this realm is the learning of protein dynamics directly from MD simulation trajectories, followed by the generation of analogous trajectories or corresponding ensembles. Conventional MD simulations proceed by numerically solving Newton's equations of motion under a predefined force field, iteratively updating the atomic coordinates of the protein system and thereby inherently capturing the temporal sequence of its evolution. However, standard all‐atom MD simulations necessitate typical integration timesteps of 1–2 fs; even with specialized techniques—such as virtual sites or hydrogen mass repartitioning—the timestep rarely exceeds 5 fs. Consequently, probing conformational changes that unfold over extended timescales incurs substantial computational costs in traditional MD. In contrast, generative AI frameworks can reduce the dependence on explicit stepwise propagation, thereby alleviating sampling challenges and enabling efficient exploration of long‐timescale dynamics [207].

AI‐based methods for conformation and trajectory generation can be broadly divided into two categories: The first modifies the inputs to existing structure‐prediction models, particularly AlphaFold, without altering their architectures. Representative examples include SPEACH_AF [208], AF‐Cluster [209], and AFsample2 [210], which generate diverse conformations by perturbing multiple sequence alignments (MSAs). SPEACH_AF introduces noise and sliding‐window masking to weaken coevolutionary constraints, AF‐Cluster partitions MSAs into similarity‐based subsets to encourage state‐specific predictions, and AFsample2 applies stochastic column masking to broaden ensemble diversity. These approaches are straightforward to implement and require no architectural redesign. The second category involves purpose‐built generative architectures, including GANs, VAEs, normalizing flows, and diffusion‐based models. Models also differ in whether they aim to sample (temporal) dynamics directly or whether they only aim to sample a conformational ensemble.

GAN‐based approaches, including idpGAN [211], DeepPath [212], and AAE‐GS [22], demonstrated that adversarial learning can generate conformational ensembles and transition pathways, particularly for IDPs and small peptides. These methods often employ multi‐discriminator or active‐learning strategies to improve structural fidelity and mitigate data limitations. In parallel, VAE‐based frameworks such as LAST, MSES, and ICoN exploit continuous latent representations to accelerate conformational exploration. LAST iteratively retrains a VAE on sampled trajectories and launches targeted simulations from low‐density latent regions, while MSES integrates VAE‐derived CVs into enhanced sampling protocols.

Despite these advances, GANs and VAEs exhibit well‐known limitations. GANs often suffer from training instability and mode collapse, whereas VAEs frequently generate overly smooth conformations due to latent regularization, compromising structural detail and sharp transitions. These shortcomings have driven the field toward flow models and diffusion models, which provide greater training stability and improved fidelity. Both rely on likelihood‐based or score‐matching objectives that avoid unstable minimax optimization, resulting in more robust convergence and improved mode coverage. Flow models provide exact likelihood estimation and invertible mappings for efficient latent‐space manipulation, whereas diffusion models progressively denoise noisy samples, enabling accurate modeling of complex multimodal conformational distributions. These advantages have made flow and diffusion models increasingly influential approaches in modern generative AI for structural biology [84, 213].

Normalizing flow and flow‐matching models, including AlphaFlow [214], P2DFlow [215], BBFlow [216], SIRAH‐Flow [217], and MDGen [21] have emerged as promising approaches for ensemble generation. These methods typically employ SE(3)‐equivariant architectures conditioned on sequence, structural, or energetic priors. AlphaFlow reformulates AlphaFold under a flow‐matching objective, transforming a deterministic structure predictor into a generative ensemble model capable of capturing experimentally observed heterogeneity. P2DFlow extends this concept by integrating ensemble‐aware latent representations and physics‐informed energy priors, improving sampling of intermediate states. BBFlow adopts a backbone‐only representation for substantially faster sampling, while SIRAH‐Flow combines normalizing flows with CG force fields to generate Boltzmann‐like distributions efficiently. And MDGen treats trajectories as “molecular videos”, learning dynamical properties such as free‐energy surfaces and Markov fluxes while achieving orders‐of‐magnitude acceleration over conventional MD.

Diffusion models have become increasingly prominent in this area due to their ability to iteratively denoise structural representations while naturally incorporating physical priors. Recent examples, including Str2Str [218], DiG [219], DeepConformer [220], ExEnDiff [221], Str2Str‐FT/NE, ConfDiff [222], ATMOS [223], AnewSampling [224], and TEMPO [225] demonstrate promising performance to generate conformational ensembles and trajectories. These models differ primarily in their training data and physical conditioning strategies. Str2Str generates ensembles directly from static structures without requiring MD trajectories. DiG incorporates both experimental structures and MD data to better approximate underlying energy landscapes. DeepConformer learns sequence‐conditioned conformational distributions from structural databases alone, recovering native‐like ensembles and fold‐switching behavior. ConfDiff explicitly integrates force‐guided diffusion dynamics, balancing conformational diversity with low‐energy fidelity. ATMOS employs state space models to efficiently generate atomistic MD‐like trajectories with linear scaling in sequence length. TEMPO utilizes a hierarchical multi‐scale autoregressive framework to capture both slow collective motions and fast local fluctuations in protein conformational ensembles.

Distinct from trajectory‐focused denoising pipelines, methods such as BioEmu and DynaFold emphasize the use of diffusion models to sample equilibrium conformational ensembles and conformational distributions of protein systems. BioEmu employs an SE(3)‐equivariant diffusion model architecture. It integrates the Evoformer module from AlphaFold2 for sequence encoding and generates structures through a diffusion process, enabling the sampling of functional motions including local unfolding, domain rearrangements, and cryptic pocket formation [226]. DynaFold follows a similar paradigm but uses a latent diffusion‐based framework, learning protein‐specific dynamic features from limited trajectory data. It achieves favorable efficiency in sampling equilibrium ensembles and dynamical transition pathways [227]. While most frameworks focus on structured proteins, specialized architectures like IDP‐Fold [228] and idpSAM [229] have extended these capabilities to the complex landscapes of IDPs.

Complementary approaches based on stochastic interpolants further expand this landscape. For instance, EquiJump [230] learns transferable large‐timestep leap dynamics that aim to preserve aspects of kinetics and native stability, while Implicit Transfer Operators (ITO) and its transferable variant (TITO) learn transition densities across multiple time resolutions from equilibrium MD data [231, 232]. This capability allows them to reduce reliance on femtosecond‐scale integration timesteps, facilitating simulations that are up to four orders of magnitude faster while aiming to preserve the Boltzmann distribution. Additional models like PTraj‐Diff [233], ConfRover [234], BioMD [235], GLDP [236], PVB [237] and PLACER [238] further demonstrate efficacy in diverse protein systems, collectively advancing AI‐driven trajectory generation toward scalable, physics‐informed biomolecular simulations. A key problem in developing such models is that they aim to sample motions beyond what is feasible with traditional MD simulations, making it difficult to obtain data to train and validate them.

Beyond direct AI‐driven trajectory generation, an alternative strategy capitalizes on protein properties implicitly learned by generative AI models, combining them with physics‐based methods to match the accuracy of all‐atom MD simulations at far lower computational cost. A prominent example is bAIes [239, 240], which uses a Bayesian framework that takes high‐confidence distance maps and AlphaFold2/3‐derived pLDDT values as prior information, with a classical all‐atom force field as the likelihood. This integration yields physically realistic conformational ensembles and greatly enhances local structural accuracy in flexible regions, disordered segments, and binding pockets.

Despite their promise, current generative models remain computationally intensive to train, often requiring massive MD datasets and substantial GPU resources. Moreover, most are optimized for soluble globular proteins under near‐physiological conditions, with limited transferability to out‐of‐distribution systems. Addressing these limitations will require the continued development of specialized conditional generative architectures tailored to challenging biological systems, including protein‐ligand complexes [221], membrane proteins [241], post‐translationally modified proteins, and proteins exposed to non‐physiological pH and temperature conditions [224, 242, 243].

#### 5.1.2 Imposing Physical Constraints on AI Models

Of course, in the context of AI‐driven trajectory generation, purely data‐driven generative models may yield physically implausible conformations or trajectories—such as an overrepresentation of high‐energy states or violations of symmetry principles. The incorporation of physical constraints can markedly improve the physical plausibility, stability, and biological relevance of the resulting trajectories. The most prevalent approach involves imposing equivariance constraints, typically achieved by employing equivariant graph neural networks (e.g., EGNN [244] or TorchMD‐NET [245]) as the backbone architecture for diffusion or flow‐matching models. This ensures compliance with the requisite SE(3) or E(3) equivariance in 3D space (invariance under rotation, translation, and reflection), thereby rendering the generated trajectories independent of arbitrary coordinate frame choices [222, 246]. Additional refinement of physical fidelity can be attained through more targeted constraints. For instance, energy‐ or force‐guided mechanisms may be introduced by augmenting the loss function or sampling procedure with terms derived from potential energy (e.g., force‐field energies or Boltzmann factors), thereby biasing the generation toward low‐energy states. Alternatively, physics‐informed neural networks (PINNs) can integrate differential equations (such as Newton's equations of motion) or explicit energy functions as regularization terms during training or inference, effectively steering the sampling process toward physically consistent outcomes (Table 2) [247, 248].

**TABLE 2:** AI‐Based Protein Ensemble or Trajectory Generation.

Methods	AI algorithm	Training data	Model Scale	Outputs
DeepPath [212]	GAN	MD	AA	Trajectories
idpGAN [211]	GAN	MD	CG	Ensembles
MSES [249]	VAE	MD	CG/AA	Ensembles
EntangledSBM [250]	Entangled Schrödinger Bridge Matching	MD	AA	Ensembles & Trajectories
trX2‐D [251]	Transformer	X‐ray & NMR	AA	Ensembles
AlphaFlow [214]	Flow model	PDB + MD	AA	Ensembles
P2DFlow [215]	Flow model	MD	AA	Ensembles
BBFlow [216]	Flow model	MD	AA	Ensembles
PepTron [252]	Flow model	PDB / MD	AA	Ensembles
IDPFold2 [253]	Flow model	PDB / MD	AA	Ensembles
MARS‐FM [254]	Flow model	MD	AA	Ensembles
TBG [255]	Flow model	PDB / MD	AA	Ensembles
HollowFlow [256]	Flow model	PDB / MD	AA	Ensembles
DeepJump [257]	Flow model	MD	AA	Ensembles
F3low [258]	Flow model	MD	CG	Ensembles
UniSim [259]	Flow model	MD / PDB	AA	Ensembles & Trajectories
BioKinema [260]	Flow model	MD / PDB	AA	Ensembles & Trajectories
SIRAH‐Flow [217]	Flow model	MD	CG	Ensembles
EquiJump [230]	Flow model	MD	AA	Trajectories
MDGen [21]	Flow model	MD	AA	Trajectories
Str2Str [218]	Diffusion model	PDB	AA	Ensembles
DiG [261]	Diffusion model	MD / PDB	AA	Ensembles
DeepConformer [220]	Diffusion model	PDB	AA	Ensembles
IDP‐Fold [228]	Diffusion model	MD / PDB	AA	Ensembles
ScoreMD [262]	Diffusion model	MD	AA	Ensembles & Trajectories
FoldingDiff [263]	Diffusion model	PDB	AA	Esembles
ExEnDiff [221]	Diffusion model	MD + Exp.	AA	Ensembles
TSS‐Pro [264]	Diffusion model	MD	AA	Ensembles & Trajectories
Metadiffusion [265]	Diffusion model	Boltz‐2	AA	Ensembles
BioEmu [226]	Diffusion model	AFDB + MD + exp	CG	Ensembles
DynaFold [227]	Diffusion model	MD	AA	Ensembles
idpSAM [229]	Diffusion model	MD / PDB	AA	Ensembles
ATMOS [223]	Diffusion model	MD / PDB	AA	Ensembles & Trajectories
AnewSampling [224]	Diffusion model	MD	AA	Ensembles & Trajectories
TEMPO [225]	Diffusion model	MD	CG	Trajectories
TITO [232]	Diffusion model	MD	AA	Ensembles
GLDP [236]	Encoder‐decoder	MD / PDB	AA	Trajectories
PVB [237]	Encoder‐decoder	MD / PDB	AA	Ensembles
PLACER [238]	GNN model	PDB	AA	Ensembles

### 5.2 AI for Physics: Advancing MD Simulations Through AI

#### 5.2.1 AI‐Assisted MD Trajectory Analysis and Dimensionality Reduction

Sampling with and subsequent analysis of MD simulations often depend on prior knowledge of the target systems. For complex biochemical processes, interpreting MD sampling results solely via visualization or analyses of manually derived low‐dimensional representations may miss subtle structural transitions, risking misjudgment of core functional mechanisms [266]. ML and DL frameworks excel at handling high‐dimensional data and unsupervised dimensionality reduction. Thus, leveraging AI to differentiate between fast and slow processes in MD simulations, and to select optimal CVs—which minimize informational redundancy while effectively capturing trajectory state transitions—is critical for elucidating complex biomolecular processes. These methods employ either traditional ML algorithms (e.g., PCA, LDA, DBSCAN, ISOMAP, t‐SNE, sketch‐map, TICA) or DL approaches (e.g., VAE, flow matching, deepLDA, deepTICA) (Table 3) [267, 268].

**TABLE 3:** AI‐Driven Algorithms for Enhanced Sampling.

Methods	AI algorithm	Handles Biased Data?	Enhanced Modes
CV redundancy removal / CV dimensionality reduction / Specific CV			
TICA [269]	Linear algebra (time‐lagged ICA)	No	Biasing in MetaD; MSM featurization
kTICA [274]	Kernel trick + linear tICA	Partial	Similar to TICA, but for nonlinear features
AMINO [278]	Mutual information + filtering (ML)	Yes	Automated CV discovery + biasing
TLC [285]	Contrastive + time‐lagged learning	Partial	Time‐lagged confounder handling + automated discovery in causal TS
GREST [277]	Girsanov reweighting	Yes	On‐the‐fly enhanced sampling in biased runs
SPIB [282]	Score‐based NNs + Info Bottleneck	Yes	Iterative biasing
FMRC [283]	Flow‐matching	Partial	CV optimization for enhanced sampling
MEMnets [286]	MLP‐based NNs	Partial	General CV learning + biasing
WT‐ASBS [287]	Wavelet transform + adaptive sparse bootstrap (ML)	Partial	Automated sparse basis selection + time‐frequency confounded feature handling
ConforMix [288]	Diffusion model	Partial	Using RMSD as the CV to enhance sampling of AF3 or Boltz‐1 models
CV‐free Methods			
MESA [289, 290]	Autoencoder	No	Direct use as CV in MetaD/US
TAE [291]	Transferable AE	Partial	Biasing with kinetic‐aware CVs
VDE [292]	Variational AE	Partial	Enhanced sampling with variational CVs
RAVE [293]	Variational AE + reweighting	Yes	Iterative reweighted biasing
LD‐FPG [294]	Generative latent diffusion + physics‐info.	Partial	Enhanced generative discovery of slow modes
LaTF [295]	Linear encoder	Yes	Biasing in linear low‐dim space
Gen‐COMPAS [296]	Generative modeling	Yes	Automated generative CV discovery + biasing correction in complex enhanced sampling
Committor Learning [297, 298]	Flow model et al.	—	Targeted sampling between states
RiD [299]	Reinforcement learning	Yes	RL‐guided enhanced sampling

Among classical methods, TICA has emerged as a robust tool for analyzing temporally correlated MD data. As a time‐lagged generalization of PCA, TICA identifies collective motions with maximal autocorrelation over a specified lag time, thereby isolating slow dynamical modes relevant for long‐timescale processes [269]. The resulting low‐dimensional representations are extensively used for constructing MSMs [270] and have been implemented in analysis packages such as PyEMMA [271], MSMBuilder [272], and Deeptime [273]. Recent nonlinear extensions, such as kernel‐TICA [274] and DeepTICA [267], have further expanded this framework by leveraging neural networks to capture complex nonlinear dynamical features in protein motions.

Building on the same variational principles, VAMPnets (Variational Approach for Markov Processes networks) and their state‐free reversible variant (SRV, with a similar architecture to Deep‐TICA) adopt end‐to‐end neural architectures to directly learn slow feature functions from time‐lagged data, enabling low‐dimensional representations of long‐timescale processes and MSM construction [275, 276]. Extending this framework further, GREST incorporates Girsanov reweighting and thermodynamic corrections to handle biased trajectories, enabling both kinetic correction and optimized CV discovery for enhanced sampling [277].

Parallel efforts have focused on reducing redundancy among candidate CVs through information‐theoretic feature selection. The Automatic Mutual Information Noise Omission (AMINO) algorithm employs mutual information‐based distance metrics to assess CV similarities, applies K‐medoids clustering to group correlated CVs, selects representative CVs from each cluster, and utilizes rate‐distortion theory principles (via an information‐theoretic “elbow method”) to determine the optimal CV dimensionality, effectively eliminating redundancy [278]. Similarly, Stock et al. model candidate CVs as graph nodes with mutual information as edge weights, then applies the Leiden community detection algorithm to identify clusters of similar CVs and extract representatives with unique information [279, 280]. Other methods combine genetic algorithms with artificial neural networks (ANNs): genetic algorithms iteratively select CV subsets for training feedforward ANNs to reconstruct atomic coordinates; subset quality is evaluated by minimizing reconstruction errors, while the algorithm concurrently optimizes network architectures to identify low‐dimensional, information‐rich CVs that capture the system's essential dynamics [281]. The state‐predictive information bottleneck (SPIB) identifies metastable states in low‐dimensional representations by simultaneously optimizing state prediction and compression over a time delay, using a time‐delayed predictive objective to emphasize slow metastable transitions, while Gaussian mixture priors regularize the latent representation and promote state separation [282].

More recent flow‐based approaches emphasize dynamical information preservation. For example, the flow matching reaction coordinate learning method (FMRC) is rooted in lumpability and decomposability principles (reformulated into a conditional probability framework) and employs a feedforward encoder and flow‐matching CNF decoders for efficient data‐driven optimization with minimal prior knowledge [283]. Benchmarked on protein folding systems, it outperforms alternatives by preserving more dynamical information in low dimensions, reducing training variance, and supporting downstream applications like MSM construction.

In addition to slow variable analysis, some methods focus on conformational state clustering, followed by free energy calculations and MSM construction. For example, the CTC method utilizes normalizing flow models to estimate conditional transition probabilities from MD trajectories, identifying conformational states as dynamical islands with extremely low escape probabilities and thereby enabling precise detection of key intermediate and transition states [284].

Together, these methods are transforming MD analysis by enabling automated identification of physically meaningful low‐dimensional coordinates, thereby improving both mechanistic interpretation and downstream enhanced sampling.

#### 5.2.2 AI‐Driven Enhanced Sampling

As noted earlier, enhancing the sampling of protein dynamics by learning CVs that capture slow system processes stands as a core paradigm in AI‐assisted MD simulations. Furthermore, a growing body of research focuses on enhancing sampling by adaptively applying bias potentials or steering trajectory evolution via AI models trained on simulation data—without relying on the learning of optimal CVs. These approaches harness AI's predictive and adaptive capabilities to explore uncharted regions of the free energy landscape more efficiently, facilitating the characterization of long‐timescale processes that remain elusive to conventional MD simulations [300, 301].

Algorithms rooted in information‐theoretic dimensionality reduction and representation learning frameworks seek to balance the retention of essential system information with the minimization of representational complexity. This enables the generation of compact, physically interpretable latent spaces that can serve directly as CVs, obviating the need for manually predefined candidates. Autoencoder‐based methods include the Molecular Enhanced Sampling Autoencoder (MESA), which maps coordinates to a low‐dimensional latent space by minimizing atomic coordinate reconstruction errors, thereby capturing principal structural features [289, 290]. The time‐lagged autoencoder (TAE) incorporates dynamical information by training the decoder to predict coordinates at a lagged time (linear TAE is equivalent to TICA) [291]. The variational dynamics encoder (VDE) combines VAEs with autocorrelation losses, optimizing reconstruction loss, Kullback‐Leibler regularization, and maximization of slow mode timescales [292]. The reweighted autoencoder for variational enhancement (RAVE) adapts to biased sampling scenarios by weighting reconstruction losses with applied bias potentials (often using linear encoder activations for enhanced interpretability) and incorporating time‐lagged and mutual information terms to further accommodate enhanced sampling protocols involving external biases [293].

Despite their shared goal of latent‐space CV discovery, these methods differ substantially in optimization strategy and applicability. Reconstruction‐driven approaches such as MESA prioritize structural fidelity but may retain fast fluctuations, whereas time‐aware models such as TAE and VDE explicitly emphasize slow collective motions. RAVE extends this framework to externally biased simulations, making it particularly well suited for iterative enhanced sampling applications.

Flow‐based free energy estimation offers an alternative route. Rather than reducing dimensionality, these methods preserve full configurational information while learning invertible mappings between complex molecular distributions and tractable latent distributions [302]. A representative example is Latent Thermodynamic Flows (LaTF) [295], which models the probability density in a latent space to enable accurate free energy calculations from both biased and unbiased samples, while facilitating accelerated sampling through learned density transformations.

Another promising direction centers on direct learning of the committor function—the probability that a given conformation reaches the product state before returning to the reactant state. The committor function varies smoothly from 0 to 1 across the reactant‐to‐product transition landscape, making it an ideal CV for rare‐event sampling [303, 304, 305]. Neural‐network approximations of the committor enable targeted biasing toward transition‐state ensembles, greatly improving sampling efficiency.

Recent examples include self‐consistent iterative procedures grounded in Kolmogorov theory for direct transition‐state sampling [297], machine‐guided path‐sampling algorithms that iteratively refine committor estimates from unbiased trajectories [303], and neural‐network‐based refinement frameworks capable of recovering multiple parallel transition pathways [298]. Compared with latent‐space methods, committor‐based approaches provide a more physically direct description of reactive events, albeit at higher computational cost.

Furthermore, more aggressive strategies employ AI to actively intervene in the simulation process itself, steering sampling toward target conformational regions. Reinforcement learning approaches, such as Reinforced Dynamics (RiD) [299], train a policy network to learn adaptive bias potentials that guide the system toward underexplored or high‐barrier regions, using exploration progress or free‐energy estimates as rewards. This enables autonomous discovery of conformational transitions in complex biomolecules without predefined CVs, substantially improving sampling efficiency for rare events. Adaptive enhanced sampling methods have also evolved with the incorporation of AI elements. For instance, reconnaissance metadynamics first identifies promising low‐energy basins from short unbiased trajectories, then adaptively deposits Gaussian bias exclusively in undersampled regions, thereby enhancing efficiency on unknown free‐energy landscapes [306].

Together, these approaches substantially accelerate conformational sampling and enhance the thermodynamic characterization of protein systems, marking a major step toward autonomous AI‐guided molecular simulation [300].

#### 5.2.3 Machine‐Learned Force Fields (MLFFs) for MD Simulations

Except for analyzing and optimizing enhanced sampling protocols, AI approaches can integrate the computational precision of QM to develop more accurate machine‐learned force fields (MLFFs; also sometimes called machine‐learned interatomic potentials), thereby enhancing the fidelity of MD simulations (Table 4) [204, 307]. Some MLFFs are kernel‐based, such as Gaussian Approximation Potentials (GAP) [308], which have evolved to handle protein fragments; however, recent advancements have shifted focus toward neural network potentials (NNPs). For instance, the Accurate Neural Network Interface for Molecular Energies (ANI) family of models has been refined through training on diverse chemical datasets, achieving transferable predictions for oligopeptides and proteins with sub‐chemical accuracy while enabling nanosecond‐scale MD simulations [309]. The high‐performance LAMMPS‐ANI implementation preserves the high accuracy of the ANI neural network potentials while significantly improving computational efficiency and scalability, enabling multi‐hundred‐nanosecond all‐atom MD simulations of solvated protein systems with several million atoms [310].

**TABLE 4:** Machine Learning Force Fields.

Methods	ML/AI algorithm	Strengths
GAP [308]	Gaussian approximation potential	High accuracy with uncertainty quantification;
ANI [309]	Accurate neural network interaction	Near‐DFT accuracy; excellent transferability; fast evaluation for small‐to‐medium molecules
SchNet [311]/CGSchNet [312]	graph neural network with continuous‐filter convolutions	End‐to‐end learning; rotation/translation invariance
TorchMD [245]	TorchMD framework	Supports hybrid classical/ML potentials; easy integration and training
Allegro [315]	Equivariant deep neural network	Strict locality enables large‐scale simulations while retaining equivariant advantages
GEMS [316]	Graph‐enhanced machine‐learning potentials	High precision for complex systems
ViSNet [319]	Equivariant geometry‐enhanced graph neural network	Scalable and accurate geometric deep learning; handles directional information effectively
AI2BMD [318]	ViSNet‐based machine learning force field	Ab initio accuracy; highly efficient for large proteins; generalizable
LiTEN‐FF [321]	scalable equivariant neural network (LiTEN)	achieves quantum accuracy in conformer optimization across systems of varying sizes; Well‐suited for drug‐related simulations
Espaloma [322]	GNN	Accurately predicts drug‐protein binding energies; enables single‐GPU training within one day; low computational cost.
AQuaRef [323]	AIMNet2	Specialized MLFF for refining water‐soluble protein structures; enables accurate determination of proton positions.
SO3LR [317]	GEMs‐like	Optimized upon the GEMs framework, suitable for a broader range of molecular systems.
PCCG [329]	Potential contrasting	Coarse‐grained machine learning force field with excellent performance in protein folding.
n2p2 [320]	Neural network potential package	Mature and well‐tested framework; high accuracy
QM/MM‐ΔMLP FF [324, 325, 326]	Hybrid QM/MM with Δ‐machine learning potential correction	Combines efficiency of semi‐empirical/MM with ML corrections for improved accuracy in large systems; incorporated in AMBER
PhysNet [43, 327]	PhysNet framework	Good generalization to larger systems; incorporated in OPENMM &CHARMM

GNN‐based models, exemplified by SchNet [311], employ graph representations of atomic interactions to capture local atomic interactions in proteins. Subsequent developments in the SchNetPack toolkit have facilitated hybrid ML‐classical simulations via integration with software such as OpenMM. A notable derivative, CGSchNet [312], adopts a CG paradigm using a bottom‐up approach, balancing computational efficiency with biophysical accuracy and making it particularly suited for exploring large‐scale protein dynamics and conformational landscapes—unlike traditional CG models (e.g., Gō‐like models) [313], which are often system‐specific or fail to adequately represent multi‐body interactions critical for realistic thermodynamics [314].

Other models incorporate additional physical constraints to further improve performance. For example, TorchMD [245] and Allegro [315] introduce tensorial features and message‐passing mechanisms to model anisotropic interactions, yielding stable MD trajectories for large proteins with errors below one kcal/mol relative to QM references. Hybrid frameworks, such as the General MLFFs for Large‐Scale Simulations (GEMS) [316] and its extension SO3LR [317], combine “bottom‐up” training on diverse chemical fragments with “top‐down” refinement on full protein structures. This strategy enables accurate nanosecond‐scale dynamics simulations of systems like bovine pancreatic trypsin inhibitor while preserving Boltzmann equilibrium distributions.

A particularly notable example is AI^2^BMD [318], which addresses one of the central limitations of conventional MLFFs: poor transferability to large biomolecular systems. AI^2^BMD achieves ab initio‐level all‐atom simulation precision at computational costs reduced by orders of magnitude by employing ViSNet [319] an equivariant graph neural network trained on density functional theory (DFT) data to model protein potential energy surfaces. Leveraging millions of dipeptide conformations during training, the framework achieves strong transferability across diverse proteins and enables all‐atom simulations of systems exceeding 10,000 atoms with near‐QM precision. Similarly, differentiable end‐to‐end MLFFs frameworks implemented in libraries such as n2p2 (a high‐dimensional neural network potential package) [320], which optimize high‐dimensional neural network potentials through active learning, have substantially reduced the computational cost of protein folding simulations while retaining high accuracy. Nevertheless, despite these advances, the computational expense of large‐scale MLFF simulations remains considerable.

Recent efforts have pursued alternative strategies aimed at improving efficiency and generalizability. LiTEN‐FF [321], represents a shift toward general‐purpose foundation force fields for protein–ligand systems, achieving near‐quantum chemical accuracy through a linearly tensorized quadrangle attention (TQA) mechanism that substantially reduces computational complexity. Complementary specialized models address separate bottlenecks: Espaloma [322] focuses on protein–ligand energetics, achieving QM‐level accuracy with training costs achievable within a single GPU‐day, whereas AquaRef [323], emphasizes ensemble refinement and structural accuracy, demonstrating strong performance in resolving atomic positions and protonation states.

Despite these advances, MLFF development remains resource intensive. Training requires extensive high‐quality QM datasets, and evaluating neural network potentials at every integration step remains substantially more expensive than conventional empirical force fields. Nonetheless, for processes where classical force fields inadequately capture subtle electronic or conformational effects, MLFFs provide promising improvements in simulation accuracy.

The increasing maturity of this field is reflected in its integration into mainstream MD platforms. Examples include AMBER (featuring the QM/MM‐ΔMLP FF developed with DeePMD‐kit) [324, 325, 326], alongside OpenMM 8 and CHARMM (incorporating PhysNet) [43, 327]. More recent MD simulation platforms, exemplified by SPONGE, incorporate deep learning techniques natively, supporting neural network‐based potentials, and also provide partial implementation of AI‐guided enhanced sampling methods [328]. Collectively, these developments underscore a broader paradigm shift toward physics‐informed, AI‐driven force fields as a next‐generation foundation for biomolecular simulation.

#### 5.2.4 AI‐Based Backmapping From CG Model to All‐Atom Protein Structures

CG models are particularly effective for investigating large protein systems or processes that require extended simulation timescales, as they significantly reduce computational cost by simplifying molecular representations. However, this efficiency comes at the expense of reduced resolution, leading to a loss of atomic‐level detail. For proteins where local structural features are closely linked to biological function, recovery of full atomic resolution is often essential to enable comparison with experimental data and to accurately compute atomistic observables [330].

Early backmapping (also termed reverse mapping or reconstruction) from CG to all‐atom (AA) representations primarily employed rule‐based or heuristic methods. These approaches typically rely on fragment library searches to select local conformations or geometrically guided assembly to build the full protein structure [331, 332]. Although capable of rapidly generating reasonably AA configurations, such methods may occasionally introduce non‐physical artifacts, such as strained dihedral angles, steric clashes, and “pierced” or “punched” aromatic rings (where atoms incorrectly traverse planar ring systems during reconstruction) [332]. With advances in AI, backmapping is now widely recognized as a classic ill‐posed inverse problem, characterized by a one‐to‐many mapping from low‐dimensional CG coordinates to high‐dimensional AA structures. Data‐driven AI methods, particularly generative models, offer substantial advantages over traditional approaches by learning complex statistical distributions from large structural databases, thereby enhancing reconstruction accuracy, chemical validity, physical plausibility, and the sampling of diverse, thermodynamically relevant conformational ensembles.

Early AI‐based backmapping models often suffered from limited transferability when addressing the intrinsically one‐to‐many nature of the CG‐to‐AA mapping problem. These approaches typically demanded large quantities of system‐specific all‐atom training data and required the training of entirely separate models for each distinct molecular class or condensed‐phase environment. Notable examples include the GAN‐based backmapping frameworks developed by Li et al. [333]. and Stieffenhofer et al. [334]. for equilibrated polymer melts and other condensed‐phase systems, the VAE combined with GNN proposed by Wang et al. [335]. for small gas‐phase molecules, and the conditional variational autoencoder method introduced by Shmilovich et al. [336], which utilized 3D voxel representations derived from continuous molecular dynamics trajectory frames. While innovative for their respective domains, these early efforts underscored the challenges of generalizability, high dependence on tailored datasets, and the need for model retraining when applied to new chemical or conformational spaces.

Another strategy seeks to decompose the backmapping problem into two sequential stages: backbone mapping followed by protein side‐chain packing, wherein the backbone atoms are first determined, and the spatial positions of side‐chain atoms are subsequently predicted. This decoupled approach is anticipated to enhance generalizability and transferability across diverse systems. Representative methods include SIDEpro [337], which employs ANN to predict side‐chain conformations based on backbone‐dependent rotamer libraries; DLPacker [338], a 3D convolution U‐net network framework designed for accurate prediction of amino acid side‐chain conformations in proteins; OPUS‐ROTA5 [339], a gradient‐based side‐chain modeling toolkit assisted by deep learning predictors for refined rotamer optimization; and AttnPacker [340], an end‐to‐end, SE(3)‐equivariant deep graph transformer that directly predicts physically realistic side‐chain coordinates from backbone geometry without relying on predefined rotamer libraries. Owing to their balance of efficiency, flexibility, and reconstruction accuracy, such two‐stage frameworks have become the prevailing strategy.

Recent state‐of‐the‐art backmapping approaches predominantly leverage diffusion models and flow‐matching models, which are particularly well‐suited for generating diverse conformational ensembles and exhibit strong transferability across protein systems. These methods are typically trained on large structural repositories such as PDB [87] and the Protein Ensemble Database (PED) [341] to capture multimodal conformational distributions effectively. Notable examples include DiAMoNDBack [342], an autoregressive denoising diffusion probabilistic model that restores all‐atom details to Cα‐only CG traces in a residue‐by‐residue manner, enabling the generation of realistic, thermodynamically consistent ensembles while maintaining high transferability due to its local conditioning and autoregressive design. Similarly, FlowBack represents a generalized flow‐matching framework that employs equivariant graph neural networks to map samples from CG prior distributions directly to chemically accurate all‐atom structures, offering efficient, high‐fidelity reconstruction with enhanced generalizability [343]. Together, these generative paradigms mark a significant advancement over earlier methods by providing superior accuracy, physical plausibility, and the capacity to sample diverse conformational states without system‐specific retraining.

## 6 Integrating Physics, Experiments, and AI for the Construction of Protein Conformational Ensembles

The integration of AI, experimental measurements, and physics‐based simulations has significantly advanced protein conformational ensemble modeling. Emerging tripartite frameworks can be broadly divided into two paradigms based on how AI is incorporated: AI‐then‐Physics, where AI provides structural priors that are subsequently refined by physics‐based methods, and AI + Physics, where AI is directly embedded within ensemble generation alongside physical constraints and experimental observables.

### 6.1 AI‐then‐Physics: Sequential Integration

**FIGURE 5:**
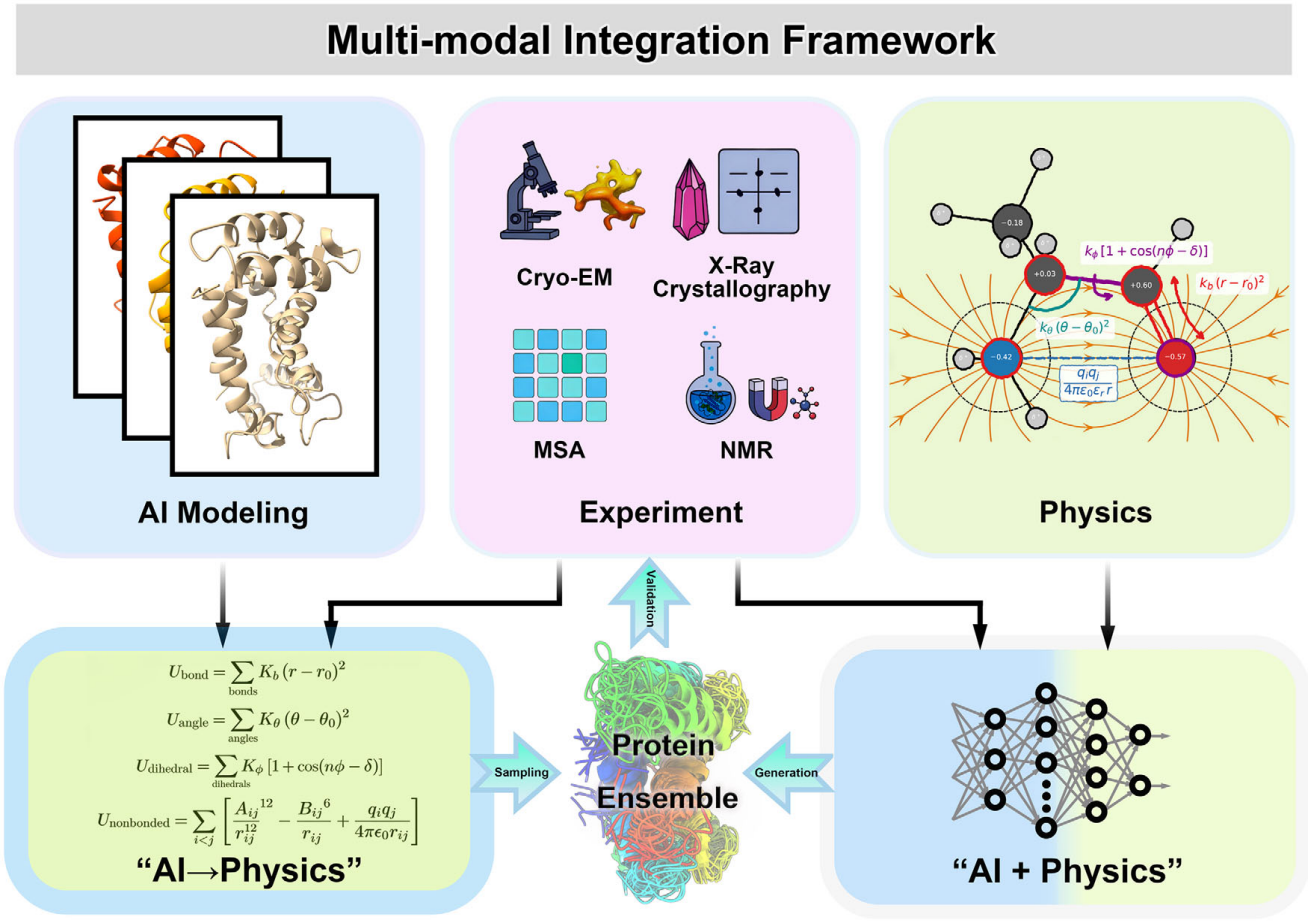
A multi‐modal integration framework for generating protein conformational ensembles, which fuses AI modeling, experimental measurements, and physics‐based sampling and force fields. It employs two synergistic pipelines: The “AI → Physics” (AI‐then‐Physics) workflow starts with AI‐predicted structures (e.g., AlphaFold outputs), which are then refined into dynamic ensembles through physics‐guided sampling, experimental restraints, and force field optimization. The “AI + Physics” (physics‐constrained AI) workflow, by contrast, embeds physical constraints directly into AI architectures, allowing AI to actively steer the sampling and inference of conformational distributions to build ensembles. Experimental data plays a flexible role across both workflows, serving as an initial input, training dataset, or final validation check. Both pathways converge to produce a comprehensive, physically coherent protein ensemble that reflects the full range of conformational dynamics.

The first and more established paradigm, termed AI‐then‐Physics (Figure 5), employs a sequential workflow that leverages the unprecedented success of AlphaFold and related AI tools in generating high‐confidence static structural models. This approach uses AI to produce initial structural predictions, which are subsequently refined and expanded into biologically relevant dynamic ensembles through physics‐based enhanced sampling, integration of experimental restraints, and force field optimization. AI therefore functions as a robust preprocessing step, establishing a reliable structural foundation for downstream dynamic modeling and refinement.

A representative example is AlphaFold2‐RAVE [344], in which a reduced‐MSA version of AlphaFold2 generates diverse starting conformations spanning broad conformational space. Short MD simulations are then used to identify slow CVs via the RAVE framework, after which metadynamics along these learned CVs accelerates the sampling of the ensemble. In this workflow, AI contributes only at the initialization stage, while final ensemble generation is driven entirely by physics‐based enhanced sampling.

Other tools exemplifying this paradigm include SAXS‐A‐FOLD [345], AlphaSAXS [346], AlphaCross‐XL [347], AF‐CALVADOS [348], DeepFRET [201], and CryoDyna [349, 350]. These methods combine AI‐predicted structures with experimental restraints and physics‐based refinement to address specific ensemble modeling challenges. For example, SAXS‐A‐FOLD enables fast ensemble modeling and optimizes the fitting of AlphaFold or user‐supplied models to SAXS data [345]; AlphaSAXS enhances the integration of SAXS constraints into AI‐predicted structures for improved solution‐state conformation modeling [346]; AlphaCross‐XL automates the mapping of XL‐MS data onto AlphaFold‐predicted structures, enabling proteome‐scale analysis [347]; AF‐CALVADOS leverages high‐confidence AlphaFold2 predictions to automatically identify and constrain folded domains, while employing the CALVADOS model to allow free conformational sampling of disordered regions, thereby enabling automated, accurate simulation of multi‐domain and intrinsically disordered proteins that faithfully reproduce experimental observables such as radius of gyration and end‐to‐end distance [348]; Tools such as DeepFRET facilitate automated classification and analysis of single‐molecule FRET data, yielding promising results in probing membrane protein dynamics [201]; and CryoDyna leverages dynamic refinement strategies to better accommodate the flexibility of proteins in cryo‐EM based initial modeling [350].

These methods may incorporate targeted refinements to better accommodate generative AI systems (e.g., AlphaFold), but overall introduce only limited modifications to the original framework. AI primarily functions as the initial component of an integrated pipeline, with subsequent ensemble generation achieved through complementary approaches (as outlined in Section 7.2). This design affords substantial flexibility at each stage, enabling extensions tailored to specific protein characteristics.

### 6.2 AI + Physics: Fully Integrated Frameworks

In contrast, the second paradigm—“AI + Physics”—encompasses a more deeply integrated, synergistic workflow that embeds AI architectures directly into the core of conformational ensemble generation, rather than treating AI as a preprocessing step. Within this framework, AI models actively collaborate with physical constraints and experimental observables to guide and bias conformational sampling, as well as to infer biologically relevant conformational distributions. While such fully integrated tripartite frameworks remain nascent, they hold promise for overcoming the inherent limitations of sequential AI‐then‐Physics workflows and delivering more predictive, mechanistic, and experimentally grounded insights into protein function.

A representative example is Cryo‐Boltz [351]. This approach employs physics‐informed diffusion posterior sampling (DPS) to incorporate experimental data—specifically cryo‐EM density maps—into a diffusion model. Through a four‐stage, multiscale guidance scheme (warm‐up→global guidance→local guidance→relaxation), the sampling trajectory is progressively steered from coarse global shapes to fine atomic details toward the ensemble distribution represented by the experimental density. Such integration highlights the distinctive advantages of AI in ensemble construction; compared to the former paradigm, it generally requires relatively lower computational resources in practical applications.

Other examples illustrate diverse integration strategies. AlphaFold‐Metainference utilizes inter‐residue distance distributions predicted by AlphaFold as restraints in parallel bias metadynamics, enabling efficient sampling of conformational ensembles for IDPs and proteins containing IDRs, with validation against SAXS data [352]; Notably, BioEmu, as mentioned earlier, exemplifies this category. Its training set integrates MD trajectories, AlphaFold predicted structures, and experimental folding free energy measurements. As a result, it achieves excellent performance in constructing equilibrium ensembles [226]. trX2‐D, an innovative output‐driven approach built on trRosettaX2 framework, integrates deep learning predictions with physics‐based iterative sampling of inter‐residue geometric distributions—first pre‐trained on high‐resolution X‐ray structures and fine‐tuned on dynamic NMR ensembles—to generate diverse conformational ensembles without relying on prior native state knowledge [251].

These approaches differ significantly in computational cost and accessibility. AlphaFold‐Metainference offers strong interpretability but remains computationally intensive. CryoBoltz is efficient once pretrained but depends on high‐quality cryo‐EM density maps. BioEmu amortizes computational cost through rapid inference after training, though model development is resource intensive. trX2‐D offers a comparatively modular and accessible framework but depends strongly on training‐data quality.

Collectively, these two tripartite integration paradigms illustrate the power of unifying physics, experiments, and AI for protein conformational ensemble construction. The established AI‐then‐Physics workflow provides a practical, efficient route to generating physically consistent ensembles from AI‐predicted structures, while the emerging AI‐plus‐Physics framework holds the potential to redefine the field through the development of fully synergistic modeling pipelines. In both paradigms, experimental data plays a flexible role—functioning as an initial input, training dataset, or final validation check. Ultimately, both approaches converge on the same goal: constructing comprehensive protein conformational ensembles that faithfully capture biological dynamics across multiple scales.

## 7 Perspective and Outlook

Dynamic biomolecules govern fundamental processes such as enzyme catalysis, ligand binding, allostery, and molecular transport, while their dysregulation is associated with diseases ranging from cancer (e.g., oncoprotein conformational switching) to neurodegeneration (e.g., amyloid aggregation). Deciphering the mechanisms of these systems requires more than isolated analytical tools or static structural snapshots; it increasingly depends on integrative frameworks combining experiments, physics‐based modeling, and AI methods. Such integration facilitates the interpretation of heterogeneous datasets in the context of protein conformational dynamics and biological function.

More fundamentally, protein function is closely linked to thermodynamic stability and conformational dynamics under physiological conditions. For conformationally coupled processes (e.g., GPCR activation, antibody‐antigen recognition), an ensemble‐based perspective is often essential: proteins populate a continuum of conformational states, ranging from relatively stable low‐energy basins that are more accessible to experiments and simulations to transient high‐energy intermediates that remain challenging to characterize directly. Experimental, physics‐based, and AI approaches each capture distinct aspects of these conformational ensembles, but their individual limitations make integrative strategies increasingly important for achieving a more complete understanding of biomolecular dynamics.

### 7.1 The Complementary Triad: Strengths, Limitations, and Integration

Experiments provide the primary experimental basis for validating computational models. Techniques including cryo‐EM, NMR, X‐ray crystallography, SAXS, HDX‐MS and others provide measurements of structural and dynamic properties—from atomic‐level interactions to ensemble‐averaged conformational shapes. Nevertheless, important limitations remain: most methods capture equilibrium state populations rather than transient and low‐population states, and ensemble averaging masks rare conformations (e.g., 1% of the total population) that contribute to allosteric regulation.

Physics‐based simulations (MD, MC) provide a mechanistic framework, applying physical principles and statistical mechanics to generate atomistic trajectories. These approaches provide access to thermodynamics (free energy landscapes) and kinetics (conformational transition timescales) that may be difficult to measure directly. Yet, they face well‐ recognized bottlenecks: the “curse of dimensionality” restricts the sampling of ms–s timescale events, while force field inaccuracies can affect the accuracy of predicted conformational ensembles.

AI has emerged as a powerful complementary approach for scalability and integration, particularly useful for high‐dimensional data analysis via dimensionality reduction and efficient predictive modeling. AI can help address several limitations of both experiments and simulations (e.g., accelerating sampling through optimal CV learning and improving aspects of force field accuracy via MLFFs). However, its performance remains constrained by limited high‐quality training data—including experimentally derived ensembles and ms‐timescale MD trajectories—remain in short supply. Furthermore, many deep learning architectures and AI‐generated conformational ensembles remain difficult to interpret mechanistically unless anchored by physical constraints.

**FIGURE 6:**
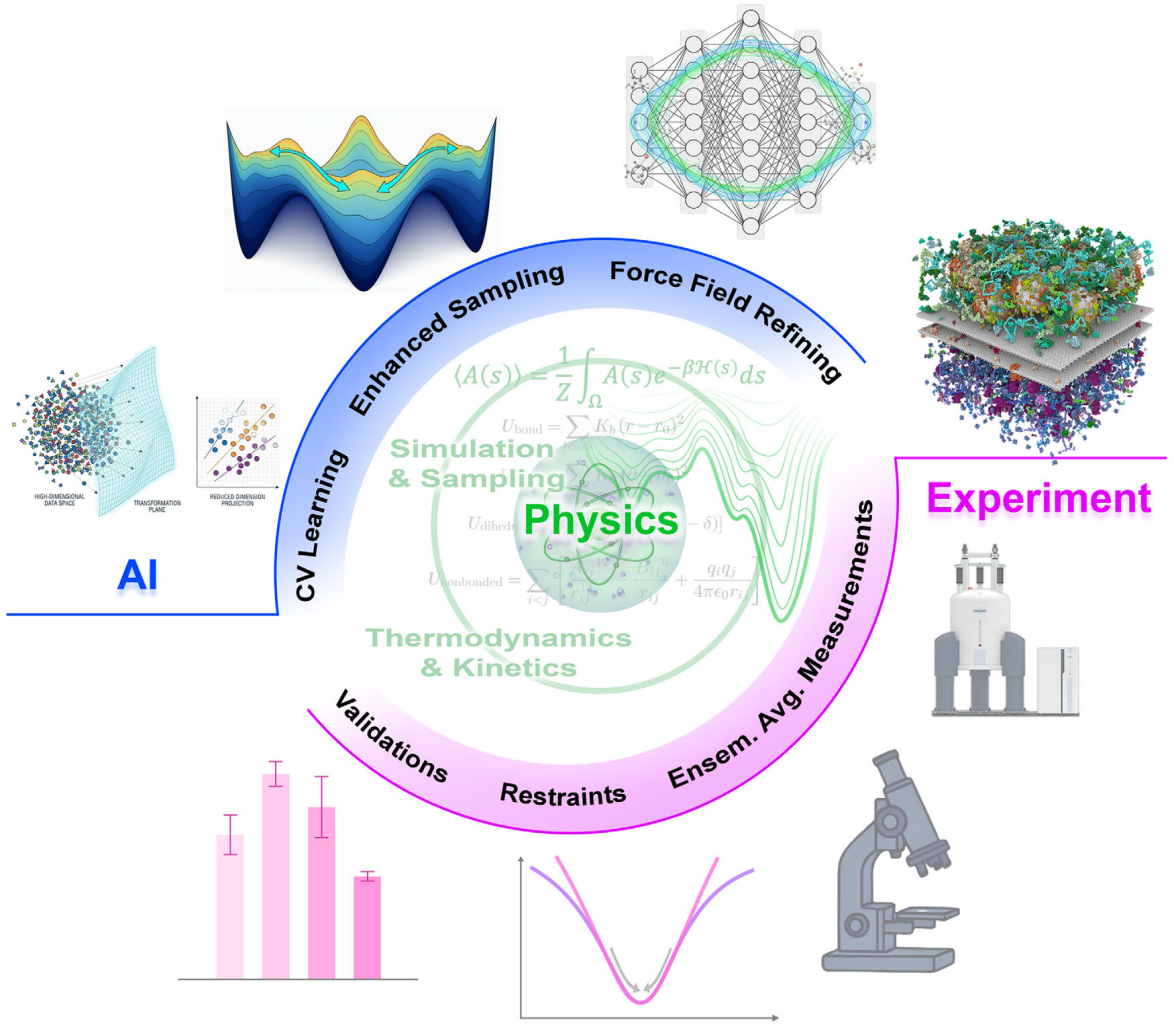
Unified, synergistic framework for integrated protein dynamic modeling, bridging experiments, physics‐based simulations, and AI. Physics acts as the integrative “glue” providing force fields and dynamic information, while experiments supply intuitive data, and AI offers reference structures and dimensionality reduction. The iterative cycle includes data collection, modeling, sampling, state analysis, and ensemble generation, with convergence checks via AI analysis.

The triad's greatest strength lies in effective integration: experiments provide empirical validation, physics ensures physical plausibility, and AI improves analytical scalability (Figure 6). Illustrative synergies include:

- Experimental restraints guide physics‐based ensemble refinement, thereby reducing the sampling of non‐physical conformations.
- AI‐derived CVs boost simulation efficiency, enabling the capture of rare conformational events while preserving important kinetic features.
- Physics‐informed AI architectures impose biophysical constraints to generate physically consistent conformational ensembles.

### 7.2 The Integrative Workflow

One possible future integrative framework could involve a data‐driven cycle that balances physical rigor with practical applicability. Its core steps could include:

1. Experimental Constraint Integration: Experimental datasets (e.g., cryo‐EM/X‐ray density maps, NMR chemical shifts, XL‐MS distances) refine initial structural models via physics‐based fitting approaches, ensuring consistency with experimental observations.
2. Enhanced Sampling: AI‐optimized CVs or experiment‐biased simulations guide physics‐based sampling to explore relevant regions of conformational space. Reweighting strategies are then employed to recover unbiased equilibrium state distributions.
3. Mechanistic Validation: AI tools analyze conformational ensembles to assess simulation convergence and identify slow collective motions, while statistical mechanics quantifies free energy differences (ΔG) associated with functionally critical transitions.
4. Iterative Refinement: Discrepancies between predicted and experimental observables trigger force field adjustments and supplementary simulations, thereby enabling iterative refinement.

This synergistic integration of methodologies is particularly useful for studying complex biological systems—such as multi‐subunit assemblies containing IDRs or those embedded in chemically heterogeneous environments—where single‐modal approaches are insufficient to capture the full range of structural and functional heterogeneity.

**FIGURE 7:**
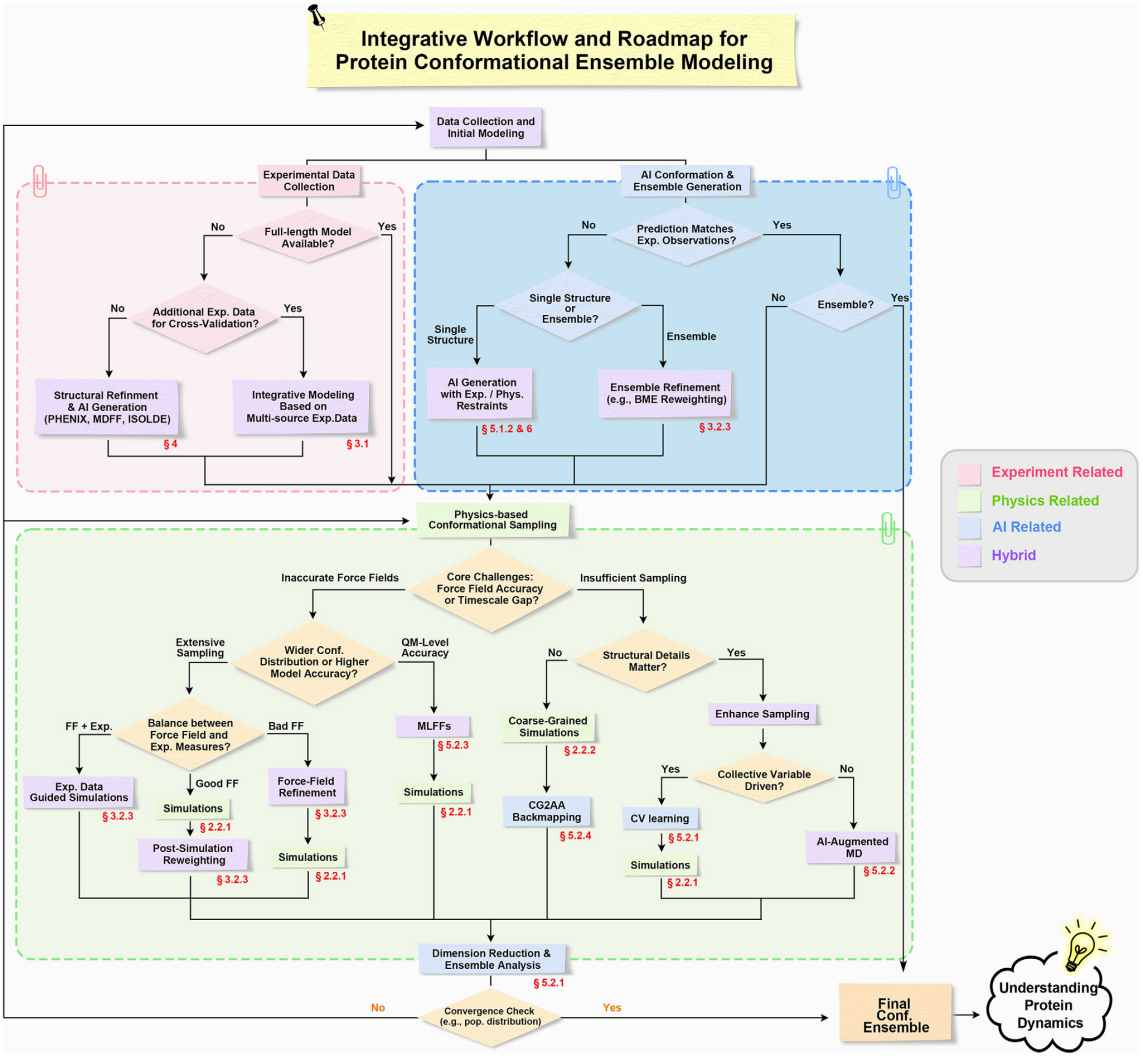
General workflow for obtaining protein ensembles and investigating thermodynamic and kinetic properties of proteins using integrated structural biology. Through data collection, sampling, AI‐driven analysis, and iterative refinement, different integration schemes can be selected based on specific research needs and emphases to combine multi‐source information, ultimately facilitating the study of protein function. Colored boxes highlight the integration methods discussed in detail in this work. The color of each box indicates the method's relationship to experimental, physics‐based, or AI approaches, with mixed colors representing techniques that integrate multiple modalities. Red annotations adjacent to the boxes denote the corresponding sections where each method is described in the text.

Operationally, this workflow can be divided into four interconnected stages (Figure 7).

The first involves initial data collection and model construction, drawing from experimental measurements, physics‐based priors, or AI‐generated structures and ensembles. Experimental structures may provide high‐resolution starting points, although flexible or unresolved regions often require completion through AI‐based modeling or hybrid reconstruction. While AI predictions offer viable starting points for systems lacking homologous templates.

The second stage is physics‐based sampling, which generates conformational ensembles with physically reasonable distributions. Two major challenges arise: force‐field accuracy and computational cost. If force‐field deficiencies stem from missing quantum effects, NNPs may be required. If inaccuracies mainly arise from parameterization, experiment‐biased simulations, force‐field refinement, or post hoc reweighting can improve agreement. When sampling efficiency is the limiting factor, CG simulations with AI‐based backmapping may suffice if local detail is less critical, whereas AI‐assisted enhanced sampling may be preferable when atomistic dynamics must be preserved.

The third stage involves ensemble analysis and validation. AI methods can rapidly assess convergence, compare ensembles against prior knowledge, and identify slow collective motions. Once convergence is achieved, free‐energy calculations and MSMs enable detailed thermodynamic and kinetic characterization.

Finally, iterative refinement addresses residual discrepancies through additional data collection or optimization of the sampling strategy.

### 7.3 Challenges and Future Directions

Despite significant advances, at least four challenges remain important areas for future development:

1. Forward Model Robustness: Integrating heterogeneous datasets (e.g., cryo‐EM + NMR + SAXS + XL‐MS) requires forward models that account for experimental noise and systematic biases. Recent innovations have improved uncertainty estimation by quantifying uncertainty, yet generalizable frameworks for multi‐modal data integration remain underdeveloped.
2. Sampling Efficiency vs. Kinetic Realism: AI‐accelerated sampling techniques can capture diverse conformational distribution related to long‐time timescale dynamics within hours of computation, but their kinetic consistency must be validated against complementary experiments. Hybrid approaches combining AI‐generated CVs with enhanced sampling methods offer a promising path forward, but require additional benchmarking against well‐characterized model systems.
3. Interpretability and Generalization: AI models must evolve beyond black‐box prediction to deliver interpretable mechanistic information. Physics‐informed architectures that encode force field terms into their loss functions enhance interpretability, yet their ability to generalize across diverse protein families (e.g., membrane proteins) and complex physiological environments remains insufficiently explored.
4. Dynamic data scarcity: Generative and dynamical AI models require large‐scale datasets, yet repositories of experimentally validated dynamic ensembles and long‐timescale MD trajectories remain limited. Data‐efficient approaches, including few‐shot learning and active learning, will be increasingly important for future progress.

Future research could prioritize applications that make these methods more robust and widely used by:

- Developing open‐access chemically and physically diverse dynamic datasets (e.g., across varying pH, temperature, and post‑translational modifications) to alleviate limitations associated with data scarcity.
- Integrating higher‐level QM more deeply into MLFFs to improve force field accuracy.
- Creating user‐friendly integrative tools that improve accessibility for experimentalists while maintaining methodological rigor.
- Advancing few‐shot active‐learning strategies and dynamic data augmentation for systems with limited conformational dynamics data.

### 7.4 Closing: Rigor in Service of Discovery

The study of protein dynamics is not solely a technical challenge, but also an important effort toward understanding the molecular principles underlying biological function. By integrating experimental approaches, physics‐based modeling, and AI methods, researchers can move beyond static or purely descriptive models to develop predictive frameworks for rational protein design, targeted drug discovery, and improved mechanistic understanding of disease processes. The convergence of these three areas represents an important methodological advance for capturing the complexity of protein conformational dynamics across multiple spatial and temporal scales, from atomic interactions to higher‐order biological functions.

The future of structural and dynamical biology will likely depend on the continued development of integrated and physically grounded approaches, in which computational models are systematically validated against experimental observations and AI predictions are guided by physical principles. Such efforts will help improve the reliability and interpretability of mechanistic models describing how protein dynamics regulate biological processes.

## Author Contributions

**Chen Shi**: writing – review and editing, visualization, conceptualization, investigation, writing – original draft. **Kresten Lindorff‐larsen**: supervision, writing – review and editing. **Yong Wang**: supervision, resources, project administration, conceptualization, writing – review and editing, writing – original draft, visualization, funding acquisition. **Peng Xiu**: writing – review and editing, funding acquisition, supervision. **Minying Low**: visualization, validation.

## Funding

This research was supported by grants from the National Science Foundation of China (32371300 to Y.W., and 12174337 to P.X.).

## Conflicts of Interest

The authors declare no conflicts of interest.

## Data Availability

The data that support the findings of this study are available on request from the corresponding author. The data are not publicly available due to privacy or ethical restrictions.
